# Rostrocaudal patterning and neural crest differentiation of human pre-neural spinal cord progenitors *in vitro*

**DOI:** 10.1016/j.stemcr.2022.02.018

**Published:** 2022-03-24

**Authors:** Fay Cooper, George E. Gentsch, Richard Mitter, Camille Bouissou, Lyn E. Healy, Ana Hernandez Rodriguez, James C. Smith, Andreia S. Bernardo

**Affiliations:** 1 Developmental Biology Laboratory, The Francis Crick Institute, 1 Midland Road, London NW1 1AT, UK; 2 Bioinformatics & Biostatistics Core Facility, The Francis Crick Institute, 1 Midland Road, London NW1 1AT, UK; 3 Human Embryo and Stem Cell Unit, The Francis Crick Institute, 1 Midland Road, London NW1 1AT, UK; 4 National Heart and Lung Institute, Imperial College London, London SW7 2BX, UK

**Keywords:** spinal cord, pre-neural progenitors, neural crest, *HOX* genes, motor neurons, neuromesodermal progenitors, NMP, human pluripotent stem cells

## Abstract

The spinal cord emerges from a niche of neuromesodermal progenitors (NMPs) formed and maintained by WNT/fibroblast growth factor (FGF) signals at the posterior end of the embryo. NMPs can be generated from human pluripotent stem cells and hold promise for spinal cord replacement therapies. However, NMPs are transient, which compromises production of the full range of rostrocaudal spinal cord identities *in vitro*. Here we report the generation of NMP-derived pre-neural progenitors (PNPs) with stem cell-like self-renewal capacity. PNPs maintain pre-spinal cord identity for 7–10 passages, dividing to self-renew and to make neural crest progenitors, while gradually adopting a more posterior identity by activating colinear *HOX* gene expression. The *HOX* clock can be halted through GDF11-mediated signal inhibition to produce a PNP and NC population with a thoracic identity that can be maintained for up to 30 passages.

## Highlights

• NMPs give rise to PNPs that undergo full, rostrocaudal diversification over time (7–10 passages)
• PNPs self-renew and can differentiate to SOX10^+^ NC with corresponding rostrocaudal identity
• GDF signaling regulates the upregulation of sacral *HOX* genes in PNPs/NC
• TGF-β signaling inhibition allows long-term stabilization of PNPs/NC in a thoracic identity

## Graphical abstract

**Figure:**
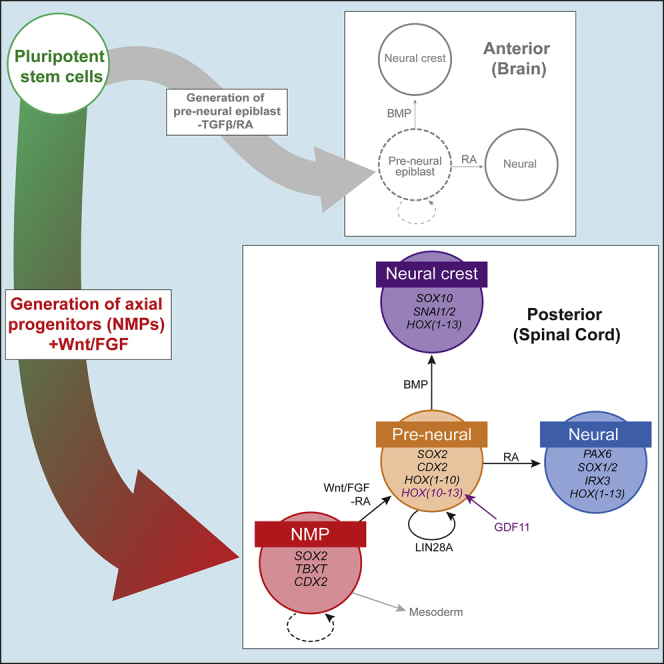

## Teaser

In this article, Cooper and colleagues describe the generation and characterization of human pre-neural progenitors (PNPs) that undergo full rostrocaudal diversification, demonstrate stem cell self-renewing characteristics, and differentiate to SOX10^+^ neural crest (NC) progenitors of corresponding rostrocaudal identity. PNPs and NC progenitors can be maintained, for up to 30 passages, in a thoracic identity by inhibiting GDF11-mediated sacral *HOX* gene expression.

## Introduction

The discovery of neuromesodermal progenitors (NMPs) as the bipotential source of spinal cord (neural) and somite (mesodermal) formation has reinvigorated efforts to generate *in vitro* models of embryonic development and disease (reviewed in Wymeersch et al., 2021). NMPs are maintained by the synergistic action of fibroblast growth factor (FGF) and WNT signals, which activate co-expression of the transcription factors Brachyury, SOX2, and CDX (CDX1, 2, and 4). Brachyury and SOX2 are mutually antagonistic cell fate determinants for the mesodermal and neuroectodermal germ layers, respectively (Gouti et al., 2017; Henrique et al., 2015; Koch et al., 2017; Tsakiridis et al., 2014; Wymeersch et al., 2016). CDX proteins act to suppress retinoic acid (RA)-mediated Brachyury inhibition (Gouti et al., 2017), activate WNT and FGF pathway components, and induce a middle *HOX* identity (Amin et al., 2016; Neijts et al., 2017; van de Ven et al., 2011). *HOX* genes are expressed in a spatial and temporal order that is colinear with their physical 3’–5’ genomic position and assign regional identity to the emerging embryonic axial tissue (Deschamps and Duboule, 2017). Colinear *HOX* gene expression is initiated by WNT signaling in the posterior streak (Neijts et al., 2017). The successive expression of 5′ *HOX* genes is induced by CDX expression but is paced by FGF signaling (Mouilleau et al., 2021; Neijts et al., 2017). More posteriorly, GDF (GDF8 and GDF11) signaling is required for *HOX10-13* gene expression and acts as part of a gene-regulatory network with *LIN28A* and *HOX13* genes regulating the proliferation of axial progenitors in the tail bud (Aires et al., 2019; Gaunt et al., 2013; Jurberg et al., 2013; Liu, 2006).

As the rostrocaudal axis elongates, NMPs that enter the pre-neural tube (PNT) downregulate *Brachyury* but maintain expression of *Sox2* and *Nkx1-2* (Diez del Corral et al., 2002; Olivera-Martinez and Storey, 2007; Storey et al., 1998). As pre-neural progenitors (PNPs) migrate into the neural tube, the switch from FGF- to RA-mediated signaling alleviates repression of the neural transcription factors *Pax6* and *Irx3* and downregulates *Nkx1-2* (Diez del Corral et al., 2003; Sasai et al., 2014; Shum et al., 1999). During this period, fate mapping and lineage tracing studies *in vivo* have suggested axial progenitors also contribute to the trunk neural crest (NC) (Wymeersch et al., 2021).

Consistent with *in vivo* evidence, combined WNT and FGF stimulation efficiently converted mouse and human pluripotent stem cells (mPSCs and hPSCs) into NMP-like cells and have since become informative in studying intricate cell fate decisions and dynamics of spinal cord and NC formation (Wymeersch et al., 2021). Neural progenitors and NC derived via an NMP intermediate have robust colinear *HOX* gene expression and represent a large range of embryonic identities along the rostrocaudal axis, often up to a lumbar identity (*HOX10-11*) (Kumamaru et al., 2018; Lippmann et al., 2015). Here we present a well-characterized and simple protocol describing the generation of PNP and NC, which acquire the full-range *HOX* identities, including the most posterior (sacral) region as determined by *HOX11-13* gene expression. Furthermore, PNPs can be stabilized by suppressing TGF-β/GDF11-mediated signaling permitting long-term culture of progenitors for at least 30 passages.

## Results

### Optimizing the generation of NMP-like cells from hPSCs through WNT modulation

Human NMP differentiation protocols differ in both the magnitude and the length of WNT stimulation, as well as with respect to the addition of other signal modulators, including FGF (Figure S1A). Several of these studies further demonstrated that the generation of posterior downstream derivatives, such as trunk NC, relies on the specification of an NMP intermediate, which occurs between a mid (3–5 μM) to high (10 μM) level of WNT signaling (Frith et al., 2018; Gomez et al., 2019a, 2019b; Hackland et al., 2019; Leung et al., 2016). To find the critical WNT signaling threshold for the generation of NMP-like cells from the WA09 (H9) human embryonic stem cell (hESC) line, we seeded cells at a fixed density (50,000 cells/cm^2^) and 24 h later exposed them to a range of concentrations (1–10 μM) of the canonical WNT agonist CHIR99021 (CHIR) while keeping the concentration of FGF2 ligands constant at 20 ng/mL for 36 h (Figure S1B). In addition, our culture medium lacked the RA precursor vitamin A (retinol) and contained the pan-RA receptor (RAR) inverse agonist AGN193109 (AGN, 10 μM) (Klein et al., 1996). RA neuralizes multipotent cells, so its degradation by CYP26A1 is essential for NMP maintenance (Abu-Abed et al., 2001; Martin and Kimelman, 2010; Sakai et al., 2001). Yet RARγ is highly expressed in NMPs, suggesting that transcriptional repression mediated by RARγ in the absence of its ligand supports NMPs and rostrocaudal axis elongation (Janesick et al., 2014). AGN additionally reduced the number of aldehyde dehydrogenase (ALDH)-positive cells by 21%, indicating that endogenous RA synthesis was significantly decreased with the addition of AGN (Figure S1C).

**Figure 1:**
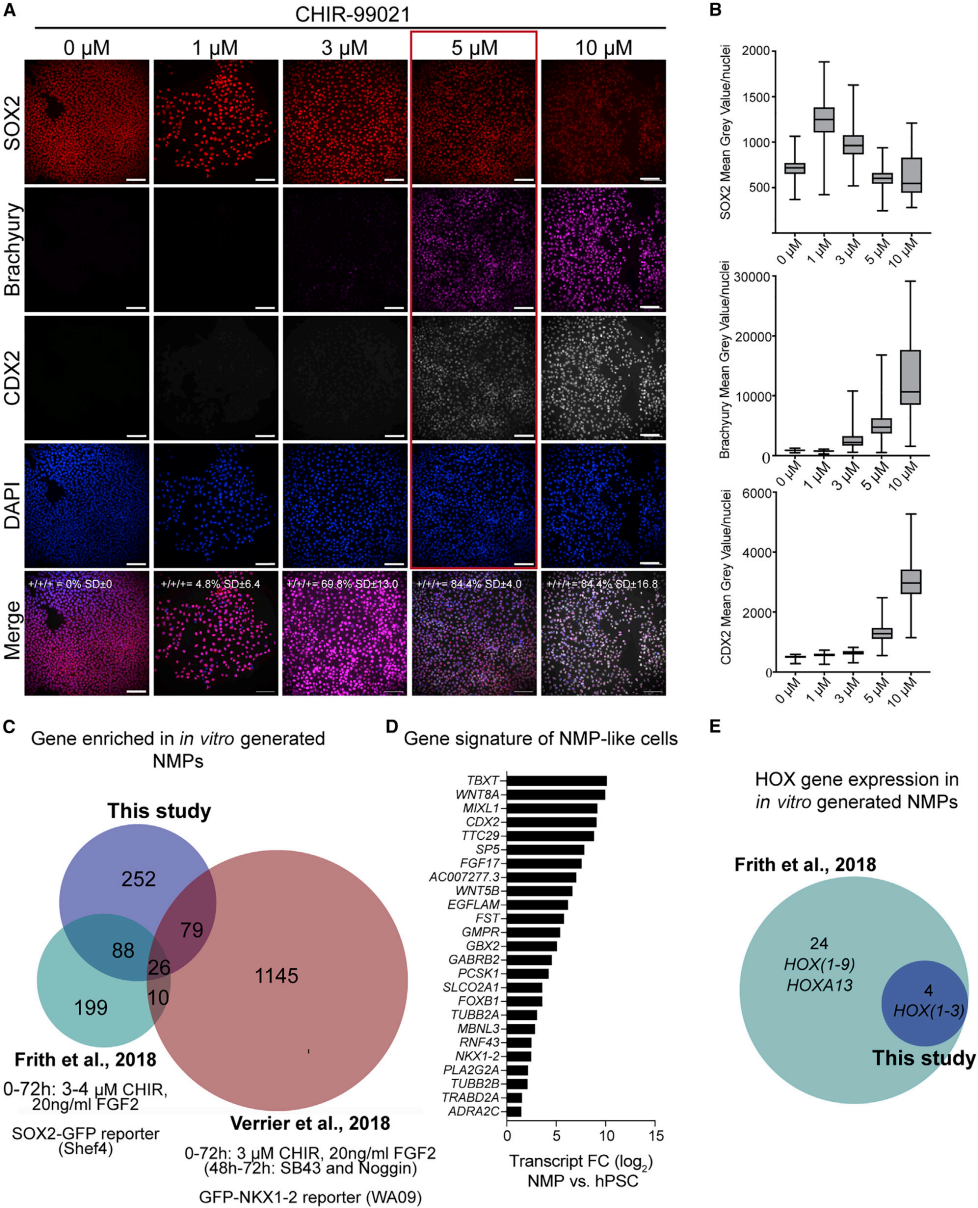
NMP-like cells are induced by intermediate WNT signaling in the presence of FGF and inhibited RA signaling (A) Representative immunostaining of 36 h cultures of SOX2 (red), Brachyury (magenta), CDX2 (gray), and DAPI (blue). Scale bars, 100 μm. (B) Boxplots showing mean gray value/nuclei quantified from repeat experiments as shown in (A). Each plot shows data points collected from two to four independent differentiations (>200 nuclei). (C) Venn diagram showing the overlap of significantly upregulated genes in NMPs as reported in this study, Frith et al. (2018), and Verrier et al. (2018). (D) Graph showing transcriptional fold change (FC) within the dataset of this study, of 26 genes commonly upregulated in NMPs according to the Venn diagram in (C). (E) Venn diagram showing the overlap of upregulated *HOX* genes in NMPs as reported in this study and Frith et al. (2018).

After 36 h, cells were analyzed for SOX2, Brachyury, and CDX2 expression by immunofluorescence (Figures 1A and 1B). Low concentrations of CHIR (0–1 μM) resulted in high expression of SOX2, while Brachyury and CDX2 were undetectable. As CHIR concentration was increased, Brachyury and CDX2 protein levels were elevated, while SOX2 expression decreased. OCT4, which is also expressed in axial progenitors and required for axis elongation (Aires et al., 2016; Gouti et al., 2017), was also lost at higher concentrations of CHIR (Figures S1D and S1E). Based on the co-expression of SOX2, CDX2, and Brachyury proteins, 5 μM CHIR (84.4% triple positive) was the optimal concentration to generate NMP-like cells from H9 hESCs at this cell density in 36 h. We could also reliably generate NMP-like cells from WA01 (H1) hESCs and the AICS-zona occludens-1 (ZO1)-GFP induced pluripotent stem cell (iPSC) line, which also required intermediate (but different) levels of WNT activation (Figures S2A–S2D). These data show that optimizing the magnitude of WNT signaling is important for obtaining NMP-like cells from different PSC lines.

### Transcriptional profiling reveals a common NMP gene set

To further characterize our NMP-like cells, we quantified WNT/FGF-induced transcriptional changes at 36 h by bulk RNA sequencing (RNA-seq) in H9 hPSCs. 1,367 genes were significantly differentially expressed between hESC and NMP stages (445 upregulated and 922 downregulated; false discovery rate [FDR] < 1%, a fold change of at least ±2, and a base mean ≥ 100) (Table S1). To define a common gene set expressed by *in vitro* NMPs, we compared our gene list of upregulated genes with two other NMP-related gene expression studies (Frith et al., 2018; Verrier et al., 2018). Although each study used different protocols and cell lines to generate NMPs (Frith et al., 2018: 72 h of FGF2/CHIR in SOX2-GFP Shef4 hESCs; Verrier et al., 2018: 72 h of FGF2/CHIR and dSMADi, 48–72 h in GFP-NKX1-2 H9 hESCs), the comparison revealed 26 genes that were consistently upregulated in all three studies (Figure 1C). Among these were well-established NMP markers, such as *WNT8A*, *FGF17*, *FST*, and *NKX1-2* (Figure 1D). Several novel genes were also identified, including *AC007277.3*, a long non-coding transcript, *TTC29*, and *EGFLAM*, all of which may be useful as NMP markers*.* Because the NMPs in this study were analyzed at 36 h, they were found to express an earlier *HOX* gene profile when compared with day 3 NMPs generated by Frith et al. (2018) (Figure 1E). Overall, these results show that our NMP-like cells, generated in an environment of depleted RA signaling, share a distinct NMP-characteristic gene signature with other hPSC-derived NMPs.

### Prolonged culture of NMPs results in loss of mesodermal potency and the emergence of epithelial SOX2^+^/CDX2^+^ colonies

**Figure 2:**
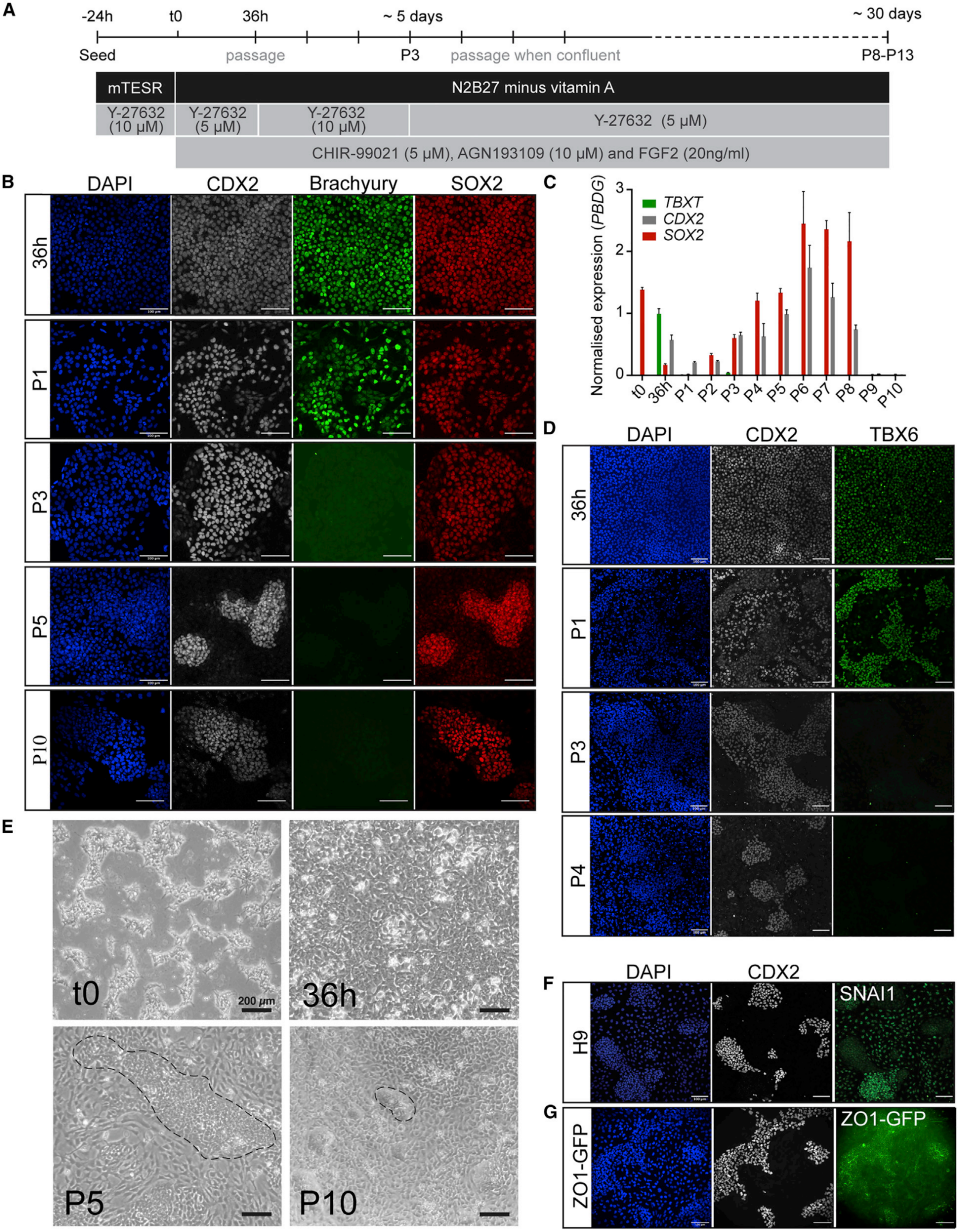
Long-term culture of NMPs in the presence of WNT/FGF and inhibited RA signaling generates epithelial SOX2^+^/CDX2^+^ cell colonies (A) Tissue culture scheme for generating NMPs and maintaining neural progenitors *in vitro*. (B) Representative immunostaining of CDX2 (gray), Brachyury (magenta), SOX2 (red), and DAPI (blue) at 36 h, passage (P) 1, P3, P5, and P10. Scale bars, 100 μm. (C) qRT-PCR analysis of NMP markers at each passage up to P10. Expression levels are normalized to the reference gene *PBDG*. Error bars show SD (n = 3 technical replicates, independent differentiations provided in Figures S3A and S3B). (D) Representative immunostaining of TBX6 (green), CDX2 (gray), and DAPI (blue) at 36 h to P4. Scale bars, 100 μm. (E) Representative bright-field images of cells at the indicated stages. Dashed lines in P5 and P10 outline examples of compact epithelial colonies, which are surrounded by flat mesenchymal cells. Scale bars, 200 μm. (F) Representative immunostaining of CDX2 (gray), SNAI1 (green), and DAPI (blue) at P5. Scale bars, 100 μm. (G) Representative immunostaining of CDX2 (gray), GFP (ZO1-mEGFP iPSC, green), and DAPI (blue) at P5. Scale bars, 100 μm.

NMPs have previously been maintained in culture for up to 7 d (Lippmann et al., 2015), but it is necessary to culture them for longer than this to generate enough cells for development of therapeutic or high-throughput assays. We sought to extend the culture of spinal cord progenitors by creating the posterior (SOX2^+^/CDX2^+^) equivalent of anterior (SOX2^+^/OTX2^+^) NSCs. To this end, we dissociated and re-plated NMP-like cells at low density at 36 h, suppressed RA signaling (by removal of vitamin A from the medium and treatment with AGN), and continued WNT/FGF treatment to minimize mesodermal commitment while halting early neural commitment (Figure 2A). During the first three passages, we noted that NMPs tended to form compacted colonies that began to detach to form floating spheres (data not shown). During these passages, 10μM Y-27632 was required to maintain cell adherence, whereas at other time points 5 μM was sufficient to maintain attachment.

Using immunofluorescence and qRT-PCR, we showed that these culture conditions maintain a SOX2^+^/CDX2^+^ cell population up to 10 passages, corresponding to ∼30 days (Figures 2B, 2C, S3A, and S3B). Similar observations were made when using H1 hESC and AICS ZO1-mEGFP iPSCs (Figures S3C and S3D). After one passage (P1) the cultures were heterogeneous, with some cells expressing the NMP-characteristic Brachyury^+^/SOX2^+^/CDX2^+^ signature. By P3, Brachyury and its immediate downstream target TBX6 were undetectable, but most cells continued to express CDX2 and SOX2, suggesting a loss of mesodermal and a maintenance of neural potency (Figures 2B–2D).

By P5, the cell population had segregated into two types, as judged by bright-field and immunofluorescence imaging (Figures 2B and 2E): one formed compact SOX2^+^/CDX2^+^ cell colonies, while the other was negative for SOX2/CDX2 and had acquired mesenchymal characteristics, such as cell spreading and SNAI1 expression (Figure 2F). The SOX2^+^/CDX2^+^ cells appeared to be epithelial, based on the accumulation of mEGFP-tagged ZO1 at tight junctions in transgenic AICS iPSCs (Figure 2G). Together, our results showed that persistent WNT/FGF signaling with suppressed RA signaling converts hPSCs via a transient NMP-like state into semi-stable epithelial SOX2^+^/CDX2^+^ cell colonies that could be maintained for 7–10 passages.

### NMPs progressively differentiate to posterior neuronal fates

To investigate gene expression changes during the transition of NMP-like cells into epithelial and mesenchymal populations, we profiled the transcriptomes of our cultures by bulk RNA-seq across 12 time points from 24 h after seeding hESCs (time point 0 h [t0]) to P10. Analysis of principal components 1 and 2 (PC1 and PC2) showed that most independent replicates (n = 3 independent differentiations) clustered together (Figure S4A). Some outliers were identified that likely reflect biological variation in our experiments. The top loading genes in PC1 (42.57% variation) included genes associated with pluripotency (*OCT4*, *NANOG*, and *EPHA1*), NC differentiation (*BMP4*, *TWIST1*, *MITF*, and *TFAP2A/B*) and posterior pattern specification (*HOX(5–13)*) (Table S2). Similarly, the top loading genes in PC2 (16.33% variation) consisted of genes associated with pattern specification, such as *HOX(1–13)*, *TBX6*, *WNT8A*, *MEIS1/2*, *FGF8*, and *CDX2* (Table S2). Gene Ontology (GO) analysis of the top upregulated differentially expressed genes in each passage was primarily associated with early embryogenesis and anterior-posterior (A-P) specification (Figure S4B).

**Figure 3:**
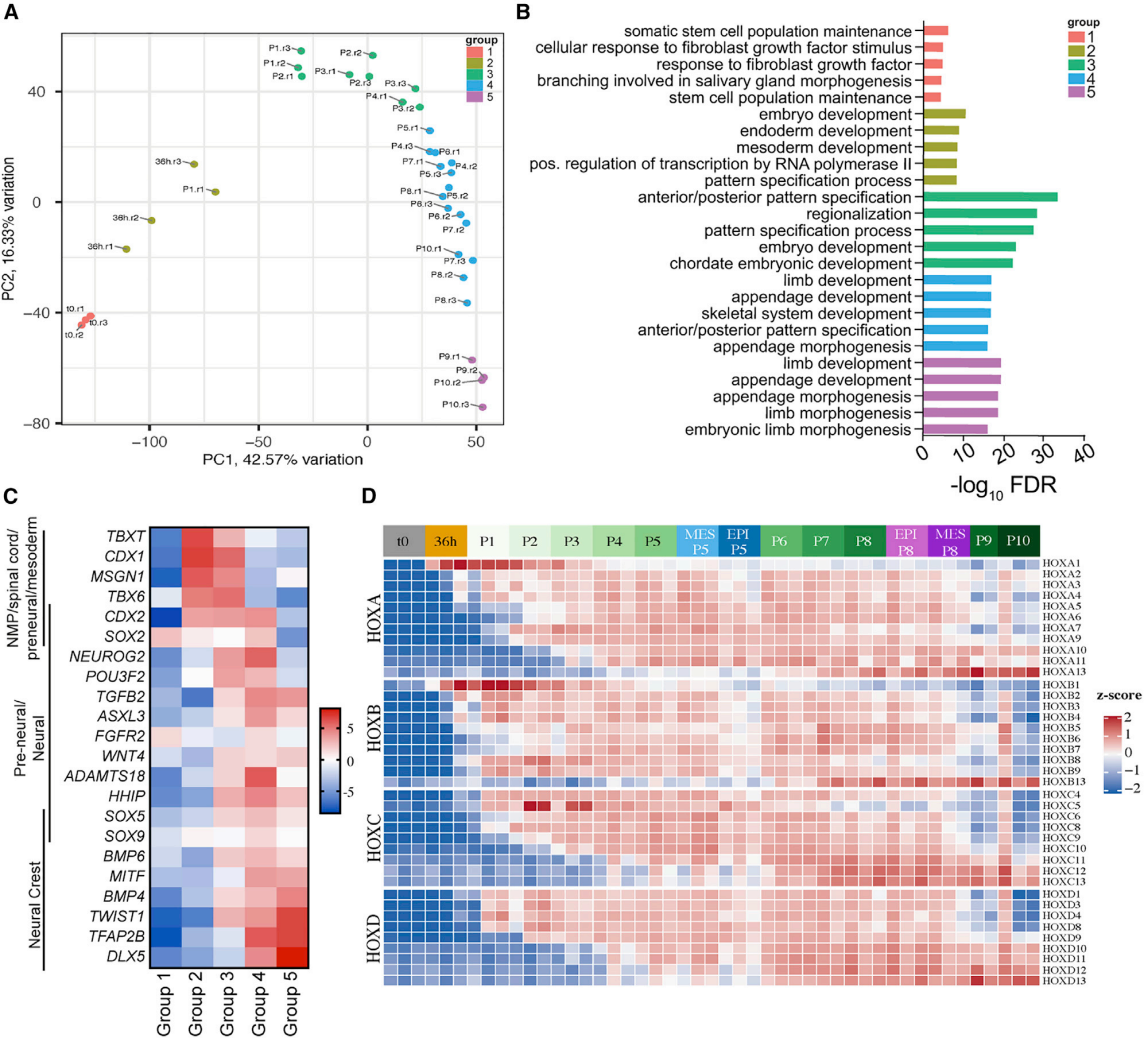
RNA-seq analysis indicates NMPs transition to neural progenitors and neural crest (NC) derivatives (A) PCA depicting variance between time points (t0 to P10) and replicates analyzed by RNA-seq. Five groups have been identified and are pseudo-colored to show grouping of replicates. (B) Top biological process GO analysis for the significantly upregulated genes in each cluster shown in (A). The corresponding Benjamini and Hochberg adjusted p values (FDR) are shown. (C) Heatmap showing FC enrichment for select NMP-, neural-, and NC-associated genes in each group shown in (A). (D) Heatmap of expressed *HOX(A–D)* genes (*Z* score) across each time point, including enriched epithelial (EPI) and mesenchymal (MES) samples at P5 and P8.

To account for biological variation between replicates and to understand specific transitions that occur over time, we categorized replicates into five groups (group 1, t0 replicates; group 2, 36 h replicates and P1.r1; group 3, P1.r2 and P1.r3, P2.r1–3, P3.r1–3, and P4.r1; group 4, P4.r2 and P4.r3, P5–P8 replicates, and P10.r1; group 5, P9 and P10 replicates) based on principal-component analysis (PCA), and a gene list defining each group was generated by comparing each group with all other groups (Figure 3A; Table S3). The top five GO biological process (GO:BP) terms associated with the top 50 upregulated genes for each group are listed in Figure 3B. Group 1 genes were associated with pluripotency. Group 2 included genes associated with germ layer specification, such as NMP-related genes such as *TBXT*, *FST*, *CDX1/2*, *TBX6*, and *MSGN1* (Figures 3B and 3C). The top genes in groups 3–5 primarily represented a change in axial identity because of being enriched for *HOX(1–9)*, *HOX(8–13)*, and *HOX(12–13)* genes, respectively (Figure 3B; Table S3). Both groups 3 and 4 showed an increase in neural progenitor markers, such as *POU3F2*, *HHIP*, *FGFR2*, and *NEUROG2*, and a reduction of some mesodermal-associated NMP marker genes (Figure 3C) (Lin et al., 2018; Olivera-Martinez et al., 2014; Verrier et al., 2018). Lastly, the top genes for group 5 contained terminal *HOX(12–13)* genes and NC-related genes, including *TFAP2B*, *DLX5*, *TWIST1*, and *MITF* (Curran et al., 2010; Frith et al., 2018; Narboux-Neme et al., 2019; Wind et al., 2021), which together suggested the cells transition to a sacral/NC identity by P10 (Figure 3C).

Next, k-means hierarchical clustering was applied to all gene-specific profiles that were significantly different over at least two consecutive time points. Each of the gene clusters showed a distinct transcriptional behavior over time (Figure S4C; Table S4). GO:BP analysis was performed for the genes in each cluster, and the most significant four GO terms are listed in Figure S4D (Table S4). Clusters 2 (C2) and 6 (C6) showed elevated gene expression from P1 to P8, when cells robustly expressed *SOX2* and *CDX2*. Consistent with the role of CDX2 in regulating colinear *HOX* gene expression (Amin et al., 2016; Neijts et al., 2017), *CDX2* and *HOX(1–9)* genes were grouped together in C2, which showed “regionalization” as the most enriched biological process. Conversely, *SOX2* was clustered with other neural fate determinants, including *SOX21*, *SP8*, and *GBX2* in C6, and thus this cluster was linked strongly with various biological functions of neurogenesis (Li et al., 2014; Luu et al., 2011; Sandberg et al., 2005). As expected, the most posterior *HOX* genes were found in C4 and C9, which showed a peak of expression around P7–P8 and P9–P10, respectively. This was in line with previous findings indicating *HOX13* genes retro-inhibit anterior *HOX* and *CDX2* transcription (Denans et al., 2015). Thus, we observed full colinear *HOX(1–13)* gene expression across 10 passages (Figures 3D, S4E, and S4G). A similar colinear *HOX* gene expression pattern was noted when using H1 hESC and AICS ZO1-mEGFP iPSCs (Figures S4H and S4I).

In parallel with the onset of terminal *HOX* expression, C1, C4, C5, and C9 included genes with elevated expression at P9–P10 (Figure S4C). These clusters were enriched for more terminal cell fates such as cell death (C1), the circulatory system (C4), axonal (C5), and skeletal/renal (C9), suggesting that cells begin to differentiate at P9 and providing a genetic explanation for the decrease in cell viability and the increase in cell spreading at late passages (Figures S4C and S4D). These results suggest that cells exit the cell cycle (C1) and upregulate genes such as *SOX9* and *NOG* (C9) and *DLX5/6*, *SOX11*, *TFAP2A*, and *BMP4* (C4), which may be indicative of differentiation into cell types such as NC. This is also consistent with the top loading genes and NC genes associated with PC1 (Table S2) and the enrichment of NC genes in group 5 passages (Figure 3C). Together, deep transcriptional profiling suggests that our NMP-like cells adopt a pre-neural fate by P4. During P4–P8 these cells remain pre-neural but progressively transit from thoracic to lumbosacral identity. At P9, cells further differentiate to a more terminal and sacral cell type, which is also enriched for NC markers.

### NMP-derived cells stabilize as epithelial PNPs and NC

To determine the extent to which NMP-derived cells undergo differentiation, we enriched epithelial and mesenchymal cells by enzymatic (TryPLE) selective detachment of the different cell types at P5, profiled by bulk RNA-seq, and compared with the original NMP-like transcriptional profile (Figure S5A). The temporal progression from 36 h to P5 accounted for most of the gene variation (PC1, ∼70%) that was detected. The lineage bifurcation of NMP descendants led to the identification of 907 differentially expressed genes between epithelial and mesenchymal cells (426 genes up in epithelial and 481 genes up in mesenchymal cells; FDR < 1%, ≥2-fold change, DESeq2 base mean > 100 reads; Table S5). Molecular function GO terms for both samples included “growth factor binding” terms, which primarily represented WNT/FGF signaling genes in addition to TGF-β superfamily signaling genes (Table S5). Both positive (*BMP4/5*, *TGFB2/3*) and negative (*NOG* and *BAMBI*) regulators of TGF-β signaling were found to be differentially expressed between P5 epithelial and mesenchymal cells, but this did not clearly indicate whether TGF-β signaling was active or inhibited in either cell type (Figure S5B; Table S5). However, epithelial cells expressed significantly higher levels of several FGF ligands (*FGF-7,-8*, *-9*, *-12*, and *-13*) and WNT receptor genes (*FZD8* and *FZD10*) (Figure S5B), whereas mesenchymal cells expressed significantly higher levels of the non-canonical *WNT11* and the canonical *WNT2B* gene and expressed significantly less canonical WNT antagonist, such as *SFRP2* and *TRABD2A*. Together this analysis further suggests that genes that modulate several signaling pathways, including TGF-β, WNT, and FGF, are differentially expressed between mesenchymal and epithelial cell types and therefore may influence cell identity and stability over time.

**Figure 4:**
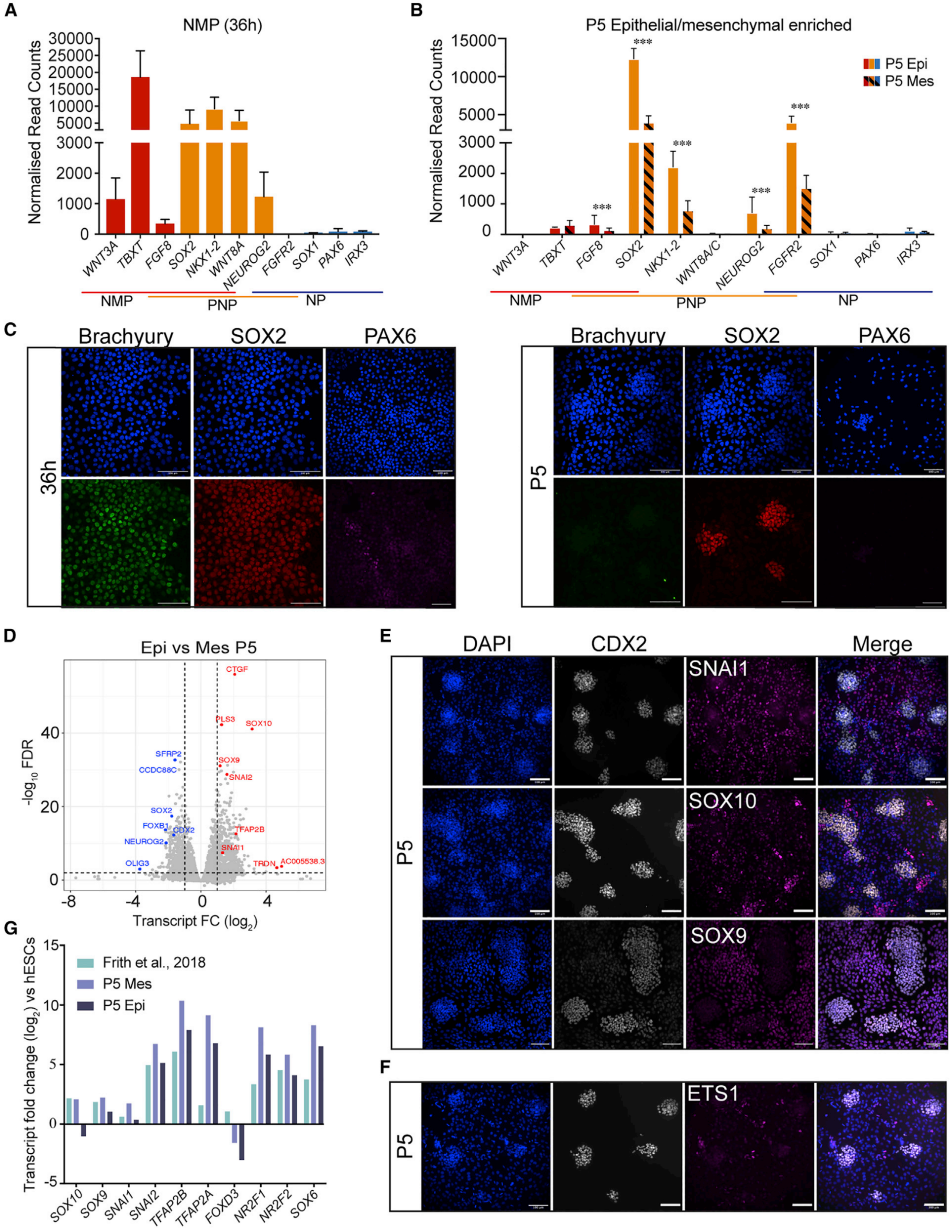
NMP-derived cells stabilize as epithelial PNPs and NC progenitors (A and B) Normalized expression levels of known markers of NMPs, PNPs, and NPs at 36 h (A) and in P5 epithelial- and mesenchymal-enriched samples (B) as determined by RNA-seq. Error bars show SEM (n = 3 independent differentiations). ^∗∗∗^FDR < 1%, a FC of at least ±2 compared with epithelial-enriched samples, and a base mean > 100. (C) Representative immunostaining of Brachyury (green), SOX2 (red), and PAX6 (magenta) confirming the expression patterns shown in (A) and (B). Scale bars, 100 μm. (D) Volcano plot showing differential expression between epithelial (blue) and mesenchymal (red) cells at P5. (E and F) Representative immunostaining of NC markers SNAI1, SOX1 and SOX9 (E), and ETS1 (F) co-stained with epithelial PNP marker CDX2 (gray) and DAPI (blue). Scale bars, 100 μm. (G) Log_2_ FC (versus hESCs) of NC marker genes in P5 mesenchymal- and epithelial-enriched samples compared with previously published trunk NC microarray data (Frith et al., 2018).

Next, a panel of previously established NMP, PNP, and neural progenitor marker genes was used to pinpoint neural progression *in vitro* (Olivera-Martinez et al., 2014; Ribes et al., 2008; Verrier et al., 2018). As expected, 36 h cells were positive for NMP markers (*FGF8*, *WNT3A*, and *TBXT*) and NMP/PNP (*SOX2*, *NKX1-2*, and *WNT8A/C*), while the NP determinants *PAX6, IRX3*, and *SOX1* were not transcribed (Figure 4A). By P5, both epithelial and mesenchymal cells had lost most NMP-exclusive expression, while the NMP/PNP markers *SOX2* and *NKX1-2* were retained and more highly expressed in epithelial cells (Figure 4B). *NEUROG2* and *FGFR2*, two PNT/NT markers, were also active in P5 cells and were significantly higher in P5 epithelial cells (Olivera-Martinez et al., 2014; Ribes et al., 2008). Furthermore, neural progenitor markers were low or absent in epithelial and mesenchymal P5 cells (Figure 4B). Immunofluorescence for Brachyury, SOX2, and PAX6 confirmed this transcriptional analysis (Figure 4C). Together, we find that epithelial colonies have a PNP identity and do not express key neural maturation genes.

We next sought to determine the identity of the mesenchymal cells. Although NMPs are known to give rise to somitic mesoderm, we did not observe Brachyury expression (pan-mesoderm marker) or its downstream target TBX6 (pre-somitic mesoderm marker) following P1 (Figures 2B and 2D), suggesting the mesenchymal cells that arise at P3–P5 are not mesodermal in origin. *In vitro* studies have revealed that NMPs can differentiate to trunk NC cells (Frith et al., 2018; Gomez et al., 2019b; Hackland et al., 2019). Moreover, our bulk RNA-seq suggested that over passaging there was an increase in genes associated with cell migration and NC (Figure 3C). Thus, we first determined whether mesenchymal P5 cells had acquired NC-specific gene expression. Transcriptome-wide analysis showed that several NC markers genes, including *SNAI1*, *SOX9*, and *SOX10*, were significantly higher in mesenchymal cells compared with their epithelial PNP counterparts (Figures 4D and S5C). This was corroborated by immunolabeling studies of SOX10, SOX9, and SNAI1 in P5 cell cultures (Figures 2F and 4E). In support of a posterior NC identity, mesenchymal P5 and P8 cells progressively expressed more posterior *HOX* genes, mirroring the PNP rostrocaudal identity (Figure 3D). By contrast, the cranial NC marker ETS1 was detectable in only a few mesenchymal cells (Figure 4F). To further validate our findings, we compared the transcript fold change of NC-related genes in both P5 epithelial and mesenchymal cells with previously published work by Frith et al. (2018) (Figure 4G). The mesenchymal cells presented in this study expressed similar or higher levels of NC markers to those presented previously by Frith et al. (2018), thereby further confirming these cells were NC in identity. Together, these results show that the mesenchymal cells surrounding PNPs are posterior NC cells and comparable with previously published *in vitro*-derived trunk NC.

### NMP-derived trunk PNPs are stem cell-like and give rise to migratory NC

**Figure 5:**
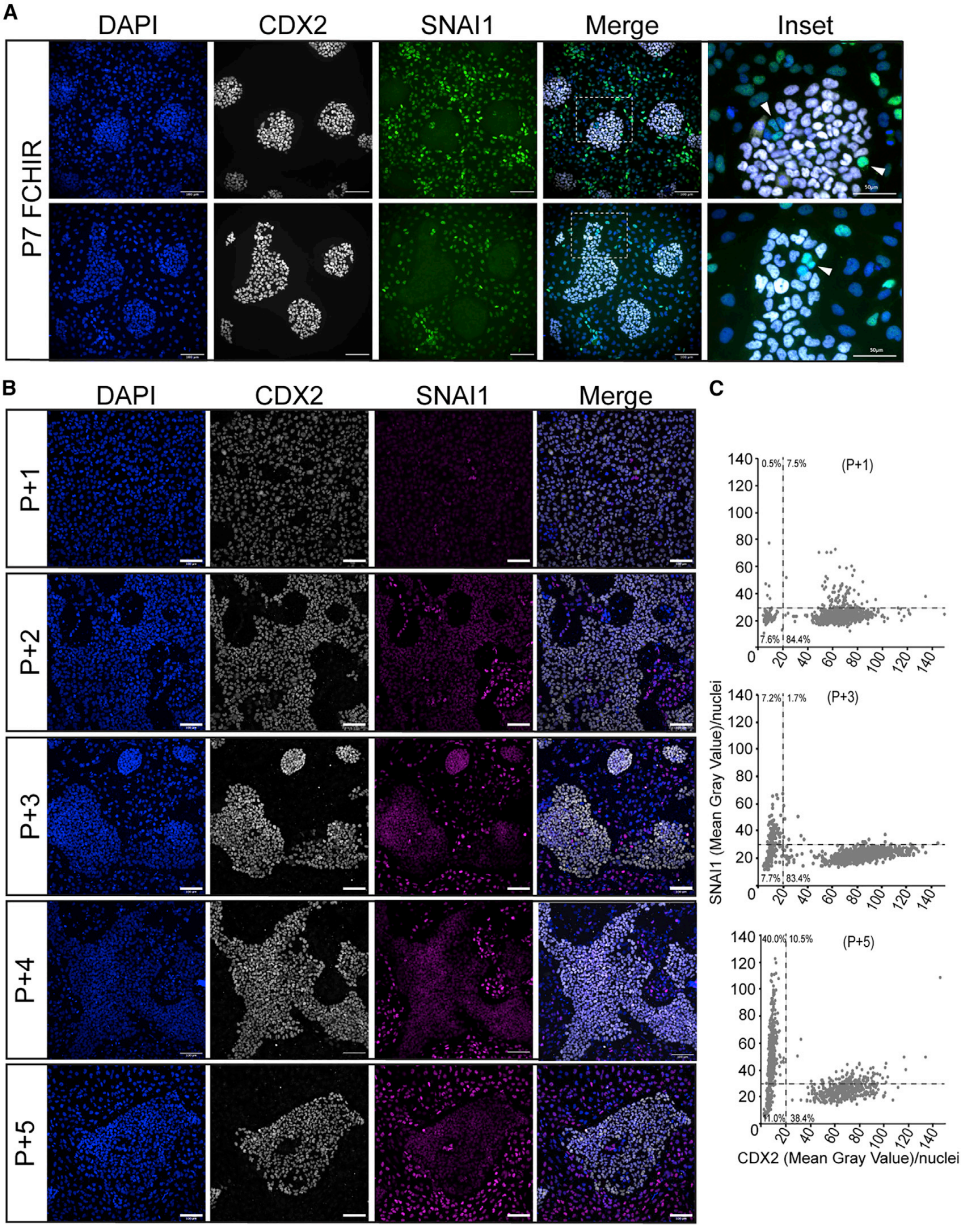
Epithelial PNPs give rise to migratory NC cells (A) Representative immunostaining of CDX2 (gray) and SNAI1 (green) co-stained with DAPI (blue) in P7 PNP/NC cultures. Inset shows magnified region identified by white dashed line, and arrow marks examples of CDX2^−^/SOX2^−^/SNAI1^+^ nuclei within PNP clusters. Scale bars, 100 μm; 50 μm (inset). (B) Representative immunostaining of CDX2 (gray), SNAI1 (magenta), and DAPI (blue) in epithelial P5 cells, which were serially passaged for four passages (P+1 to P+4) following selective detachment enrichment. (C) Dot plot showing the mean gray value/nuclei of CDX2 and SNAI1 at P+1, P+3, and P+4 panels shown in (B). Each graph shows >900 nuclei.

The immunofluorescence analysis of PNP/NC cell cultures revealed that some nuclei found within tightly clustered PNP colonies were negative for CDX2 but positive for SNAI1 (Figures 2B, 2F, and 5A), suggesting that they are undergoing epithelial-to-mesenchymal transition (EMT) and becoming NC cells (Cano et al., 2000). PNP colonies (CDX2^+^/SNAI1^−^) purified from NC cells using selective detachment were sub-cultured for four passages (P+1 to P+4) to test this idea (Figure 5B). Immunofluorescence staining showed that despite the low percentage of SNAI1^+^ NC (8%) cells in P+1 cultures, by P+4 40% of the cells were CDX2^−^/SNAI1^+^, suggesting that PNPs undergo EMT to generate NC cells (Figures 5B and 5C). Analysis of PNP (*CDX2* and *SOX2*) and NC markers (*SNAI2*, *SOX10*, and *SOX9*) by qRT-PCR in enriched cells at P+4 further confirmed this conclusion (Figure S6A). To exclude the possibility that after PNP purification, the remaining NC cells repopulate the culture over passaging, single cells from the PNP- or NC-enriched samples were re-plated by fluorescence-activated cell sorting (FACS) into single wells (Figure S6B). No colonies arose from single NC cells, suggesting that these cells have limited proliferative capacity. By contrast, single PNPs gave rise to clonal cell lines, which consisted of epithelial colonies (CDX2^+^/SOX2^+^) and surrounding mesenchymal cells (Figures S6C and S6D). Thus, the PNPs showed stem cell-like behavior by undergoing self-renewal and differentiating into NC cells.

### Modulation of TGF-β and Sonic Hedgehog (SHH) signaling locks in PNP rostrocaudal axis information by preventing GDF11-mediated sacral *HOX* gene expression

**Figure 6:**
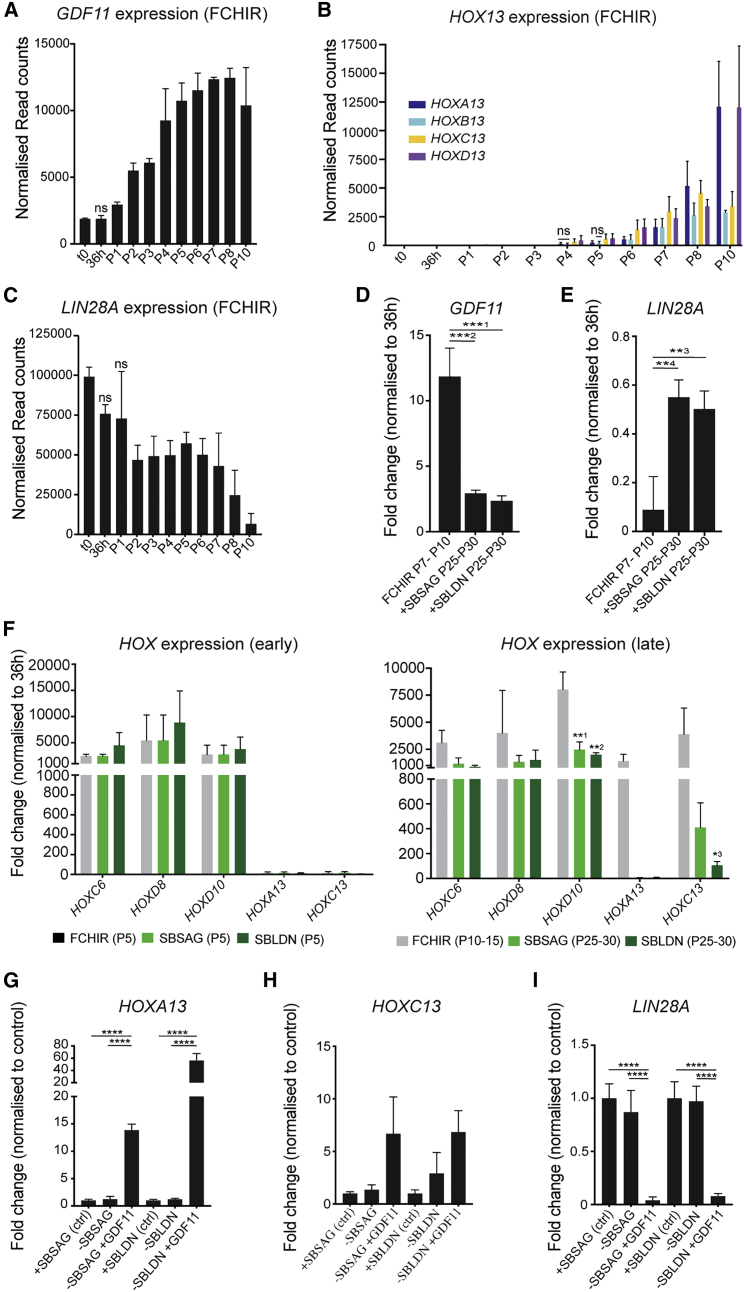
Modulation of TGF-β and SHH signaling locks in A-P information (A–C) Normalized expression levels of *GDF11* (A), *HOX13* (B), and *LIN28A* (C) at each passage as determined by RNA-seq. Error bars show SEM (n = 3 independent differentiations). All time points were called significantly differentially expressed (FDR < 1%, a FC of at least ±2 compared with t0, and a base mean > 100) unless indicated by “ns.” (D and E) Transcriptional quantification (qRT-PCR) of *GDF11* (D) and *LIN28A* (E) shown by FC over 36 h and normalized to the reference gene *PBGD* in late passage PNPs. Error bars show mean with SEM (n = 3 independent differentiations). ^∗∗1^p = 0.0003, ^∗∗2^p = 0.0002, ^∗∗3^p = 0.0026, ^∗∗4^p = 0.0045 (ANOVA, followed by Fisher’s least significant difference [LSD] multiple comparisons test [MCT]). (F) Graphs showing the transcriptional quantification (qRT-PCR) of selected *HOX* genes at early (P5) and late passages (P10–15 or P25–30) in all conditions, ^∗∗1^p = 0.0039, ^∗∗2^p = 0.0020, ^∗3^p = 0.0378 (ANOVA, followed by Fisher’s LSD MCT). Expression levels are presented as FC over the 36 h time point and were normalized to the reference gene *PBGD.* Error bars show mean with SEM (n = 3 independent differentiations). (G–I) Transcriptional quantification (qRT-PCR) of *HOXA13* (G), *HOXC13* (H), and *LIN28A* (I) in +SBLDN or +SBSAG (ctrl) conditions compared with either +SBLDN or +SBSAG without SB, LDN, or SAG (−SBLDN/−SBSAG) or with GDF11 alone (−SBLDN/−SBSAG + GDF11). Expression levels normalized to the reference gene *PBGD.* Error bars show SEM (n = 3 independent differentiations). ^∗∗∗∗^p < 0.001 (ANOVA, followed by Fisher’s LSD MCT).

We have shown that the combined modulation of WNT/FGF and RA signaling generated posterior PNPs. However, transcriptomics and lineage analysis indicated that PNP maintenance may be compromised by NC bifurcations, the progressive activation of more posterior *HOX* genes, and late-passage differentiation/cell death. In line with this, a known regulator of trunk-to-tail transition and terminal *HOX* induction, *GDF11*, was found to be significantly upregulated from P1 and increased by approximately 6-fold by P9 compared with t0 (Figure 6A). Increased *GDF11* expression precedes activation of the terminal *HOX13* genes with the onset of *HOX13* genes coinciding with a 4.6-fold increase of *GDF11* at P4 (Figure 6B). *LIN28A* was significantly downregulated at P2 (versus t0), but remained unchanged from P2 to P8, indicating that although it was decreased in expression compared with pluripotent stem cells, it remained at a sufficient level to maintain PNP cell proliferation for several passages. By P10, when PNP proliferation and culture viability were dramatically reduced, *LIN28A* was decreased over 600-fold (versus t0) (Figure 6C). With this in mind, inhibitors of Activin/Nodal (SB431542 [SB]) and BMP (LDN193189 [LDN]) signaling were used to suppress progressive posteriorization driven by GDF signaling and BMP-mediated trunk NC specification (Aires et al., 2019; Frith et al., 2018; Gomez et al., 2019b; Hackland et al., 2019; Jurberg et al., 2013; McPherron et al., 2009) (Figure S7A). Furthermore, to mimic signals that arise from the notochord during neural tube folding/cavitation and induce a ventral identity in differentiated neuronal cultures, we used a smoothened agonist (SAG) to stimulate SHH signaling (Jessell, 2000; Sasai et al., 2014).

The combined addition of SB and LDN (+SBLDN) or SB and SAG (+SBSAG) at P3 resulted in stabilization of PNPs for over 30 passages (90 days) (Figures S7B–S7D). The addition of small molecules from P3 onward did not compromise the formation of CDX2^+^/SOX2^+^ PNPs when analyzed at P5/P6 (Figures S7C and S7E). However, both supplemented conditions modestly increased the percentage of SOX2^+^/CDX2^+^ cells as quantified by flow cytometry in late passages (P9/P10) (Figure S7F). Cells maintained in +SBSAG and +SBLDN had significantly prolonged *CDX2* and *SOX2* gene expression for up to 30 passages (Figure S7B). As expected, in comparison with P7–P10 FCHIR-generated cells, *GDF11* expression was lower in +SBSAG and +SBLDN cultures (Figure 6D). In line with this, *LIN28A*, known to be downregulated in response to *HOX13* expression, was considerably reduced in FCHIR cultures by P7–P10 (Aires et al., 2019) (Figure 6E). Based on the transcriptional profiling of *HOX* genes, the positional value of the PNPs was locked at the thoracic-lumbar identity (Figure 6F). To test whether GDF11 addition, after long-term TGF-β inhibition, can induce sacral *HOX* expression, we added exogenous human recombinant GDF11 to P28–P30 cultures for 48–72 h. Short-term treatment of GDF11 was sufficient to induce *HOXA13* and *HOXC13* gene expression and suppress *LIN28A* expression (Figures 6G–6I). Furthermore, in our long-term cultures, the RA target *PAX6* remained silent in +SBLDN or +SBSAG addition at P6/P7 (Figure S7G). These results therefore show that PNPs can be locked in a thoracic identity and grown in culture for long periods of time via the addition of TGF-β inhibitors to prevent the GDF11/LIN28A-mediated transition to sacral *HOX* gene expression.

### PNPs can give rise to spinal cord neurons

**Figure 7:**
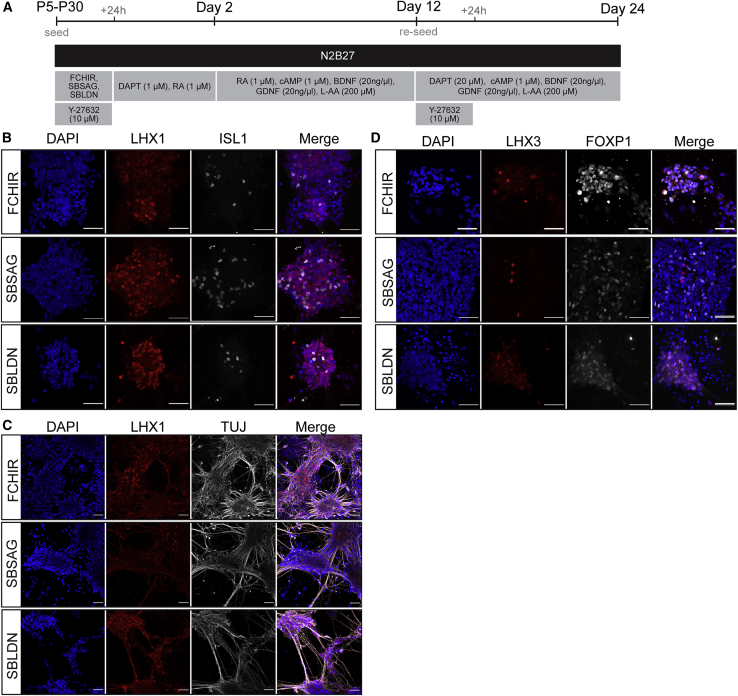
PNPs can be differentiated into neural derivatives (A) Scheme for generating differentiated neuronal cultures. (B–D) Representative immunostaining of differentiated neuronal cultures showing (B) LHX1 (red), ISL1 (gray), (C) LHX1 (gray) paired with βIII-tubulin (TUJ, green), or (D) LHX3 (red) and FOXP1 (gray). Scale bars, 100 μm.

To establish the neuronal potential of RA-deprived PNPs, we terminally differentiated P5 FCHIR and P25 +SBSAG/+SBLDN long-term PNPs into neurons (Figure 7A). Analysis of lateral motor column (LMC; FOXP1), dorsal interneuron/LMC marker (LHX1), and medial motor column markers (MMC; LHX3) found that all PNP conditions preferentially generated LHX1^+^/TUJ^+^ cells, although they did not express ISL1 (Figures 7B and 7C). The presence of LHX1^+^/ISL1^−^ neurons suggests that neurons may be lateral LMC (LHX1^+^/ISL2^+^), interneurons of the p2-dp2 domains, or medial LMC that no longer expresses early motor neuron markers (Francius and Clotman, 2014) (Figure 7B). Few cells were found to express LHX3, indicating cells preferentially differentiate MMC motor neurons (Figure 7D). Furthermore, more CHX10^+^ cells were noted in +SBSAG PNP-derived cultures, suggesting SHH signaling may introduce a more ventral identity after differentiation, giving rise to V2a interneurons (CHX10^+^/TUJ^+^) (Figure S7H) (Clovis et al., 2016). Together, these results show that our PNPs can generate various spinal cord derivatives demonstrating neuronal potential.

## Discussion

We show that with sustained WNT/FGF signaling and RA inhibition, PNPs undergo colinear *HOX* expression while transitioning to a pre-neural fate. RA inhibition prevents the upregulation of RA-responsive neural determinants genes such as PAX6, preventing neuronal differentiation. Furthermore, PNPs undergo “self-renewal,” because of high *LIN28A* and low *HOX13* expression, until increased GDF11 signaling results in an upregulation of sacral *HOX* expression. The addition of TGF-β inhibition combined with BMP inhibition or SHH agonism (+SBLDN/+SBSAG) prevents *GDF11* upregulation and subsequent loss of *LIN28A*, resulting in stabilization of PNPs in a thoracic identity for up to 30 passages. Finally, PNPs give rise to NC with a diverse range of positional axial identities, ranging from thoracic to sacral. However, the distinct differentiation potential at each axial position requires further investigation.

During development, the RA-synthesizing enzyme *Aldh2a1* is expressed in the primitive streak and in node cells (Ribes et al., 2009). Furthermore, the RARy receptor and the RA-degrading enzyme *Cyp26a1* are highly expressed in NMPs, together indicating that finely balanced RA signaling is required to regulate axis elongation (Gouti et al., 2017; Sakai et al., 2001). Moreover, loss of *Aldh2a1 in vivo* and *in vitro* results in shortening of the A-P axis and impaired NMP specification, respectively (Cunningham et al., 2015; Gouti et al., 2017; Niederreither et al., 1999). However, *Aldh2a1*-null mice produce up to 20 somites and a region of spinal cord, indicating some NMPs are specified in the absence of RA signaling; therefore, it remains unclear whether active RA signaling is essential for NMP commitment or if it acts only to maintain an expanding NMP population by regulating the *Fgf8* expression domain (Cunningham and Duester, 2015; Diez del Corral et al., 2003). Here we show that NMP specification is unaffected by the addition of a pan-RAR inverse agonist and by vitamin A withdrawal, suggesting that minimal to no RA signaling is sufficient for human NMP specification *in vitro*. Furthermore, we demonstrate that NMPs committed to a pre-neural identity, despite depleted levels of RA signaling, suggesting pre-neural commitment may also occur independently of active RA signaling. Conversely, RA depletion prevented the upregulation of definitive neural markers and neural commitment allowing PNPs to remain unfixed in their A-P identity. As a result, sustained culture (in the presence of WNT/FGF and depleted RA levels) permitted complete sequential HOX gene activation over time.

The inhibition of TGF-β and stimulation of SHH signaling during PNP differentiation was found to reduce NC delamination and to promote the stabilization of PNP cultures with a thoracic HOX signature for at least 30 passages. Specifically, our data indicate that ALK4, ALK5, and ALK7 inhibition by SB acts to prevent GDF11 signaling and is sufficient to promote PNP identity and viability in our culture by maintaining *LIN28A* expression, a key factor for the proliferation of tail bud (Andersson et al., 2006). Because cells maintained a stable progenitor identity, the thoracic *HOX* signature was not locked, and supplementation with GDF11 promoted their release to a later *HOX* signature, in keeping with its known role in regulating sacral *HOX* gene expression (Aires et al., 2019). Similarly, heterochronic grafting experiments in chick found that axial progenitors can revert from a late HOX to an earlier HOX signature, supporting the finding that *HOX* gene expression is not locked until the cells terminally differentiate (McGrew et al., 2008). This is also in keeping with *in vitro* studies that suggest that prolonged WNT/FGF signaling allows cells to reach a more posterior identity that can be fixed by inducing neural differentiation through exogenous RA addition (Kumamaru et al., 2018; Lippmann et al., 2015; Wind et al., 2021).

Our work also established that PNPs undergo EMT to form NC cells with corresponding rostrocaudal identity. Recent studies have indicated that cranial NC arises in a neural-independent manner (Leung et al., 2016). Conversely, trunk NC arises from NMPs in a BMP-dependent manner (Frith et al., 2018; Gomez et al., 2019b; Hackland et al., 2019). Here we show trunk NC progenitors are specified following commitment of NMPs to a pre-neural identity. Following this commitment, PNPs express progressively more posterior *HOX* genes over time whilst giving rise to NC with a sacral identity. Together this work suggests that the CNS and derivatives of trunk/sacral NC (such as the peripheral nervous system) arise from a common PNP derived from the NMP population. This finding has recently been supported by studies *in vivo* (Lukoseviciute et al., 2021). Surprisingly, the addition of the BMP inhibitor LDN did not prevent NC specification in long-term PNPs, although only intermediate levels of BMP signaling are required to robustly induce NC commitment (Frith et al., 2018; Hackland et al., 2017). Furthermore, the addition of the ROCK inhibitor (Y-27632) in our protocol was required to maintain a 2D culture system and has previously been shown to favor NC commitment from hPSCs; therefore, it may also play a role in promoting NC commitment from PNPs (Kim et al., 2015). Further work to test these interesting findings is required.

In conclusion, our protocol provides a valuable source of PNP and NC cells that reflect axial anterior-to-posterior progress and may hold the potential for drug screening, detailed disease modeling, or therapeutic applications. Moreover, our model provides a robust *in vitro* platform to study cellular commitments and transitions within the developing human spinal cord at greater detail.

## Experimental procedures

### Human pluripotent stem cell culture

hESCs (WA09 and WA01; WiCell) and iPSCs (AICS-23; Allen Institute) were maintained on Corning Matrigel Growth Factor Reduced (GFR) Basement Membrane Matrix (354230; Corning Incorporated) and grown in mTESR1 (85850; STEMCELL Technologies). Cells were passaged as aggregates at a ratio of 1:10/15 using Gibco Versene Solution (15040066; Thermo Fisher Scientific) (UK Stem Cell Bank steering committee approval number: SCSC13-03). Further details are available in the supplemental experimental procedures.

### NMP differentiation and PNP long-term culture

Human ESCs or iPSCs were differentiated to NMPs as described in the text. NMPs were passaged at 36 h using TrypLE express (Thermo Fisher Scientific) and when confluent thereafter. Cells were passaged as single cells at a ratio of 1:6 into NMP differentiation medium, supplemented with 5–10 μM Y-27632 (Tocris) for up to 8–12 passages. To prevent A-P axis progression, we added 2 μM SB (SM33-10; Cell Guidance Systems) and 100 nM LDN (SML0559-5MG; Sigma-Aldrich) or SB (SM33-10; Cell Guidance Systems) and 500 nM SAG (566660-1 mg; Sigma-Aldrich) to NMP differentiation medium at P3. Further details are available in the supplemental experimental procedures.

### Neuronal differentiation

Neurons were generated using a modified protocol based on a previously published neural differentiation protocol (Lippmann et al., 2015) and described in the text. Further details are available in the supplemental experimental procedures.

### Flow cytometry and immunofluorescence

Detailed experimental procedures are available in the supplemental experimental procedures.

### RNA extraction, cDNA synthesis, and qPCR

Total RNA extraction was completed using RNeasy mini kit (74106; Qiagen) following the manufacturer’s instructions. cDNA was synthesized using Maxima First Strand cDNA Synthesis Kit for qRT-PCR with dsDNase (K1672; Thermo Fisher Scientific) following the manufacturer’s instructions with the addition of a dilution step where cDNA was diluted 1:60 in water. qPCR analysis was performed using primers detailed in Table S7 on a Roche LightCycler 480 II (Roche Holding AG) using LightCycler 480 SYBR Green I Master mix (04887352001; Roche Holding AG). Relative expression was calculated using the ΔCt method, normalizing each gene to porphobilinogen deaminase (PBGD) levels.

### RNA-seq and analysis

Further details are available in the supplemental experimental procedures.

## Author contributions

F.C.: conceptualization, validation, methodology, investigation, formal analysis, writing – original draft preparation, supervision, and project administration. G.E.G.: conceptualization, methodology, investigation, supervision, project administration, and writing – review & editing. R.M.: software, methodology, formal analysis, and writing – review & editing. C.B.: investigation, writing – review & editing. L.E.H.: methodology, investigation, resources, and writing – review & editing. A.H.R.: investigation. J.C.S.: conceptualization, writing – review & editing, supervision, and funding acquisition. A.S.B.: conceptualization, methodology, investigation, writing – review & editing, supervision, project administration, and funding acquisition.

## Conflicts of interest

The authors declare no competing interests.

## Data Availability

Data are available at the GEO repository (accession number GEO: GSE150709).

## Supplemental information

Document S1. Supplemental experimental procedures, Figures S1–S7, and Tables S6 and S7

Table S1. List of genes significantly upregulated and downregulated at 36 h (NMP) compared with undifferentiated hESCs, including fold change and FDR values

Table S2. List of top loading genes for PC1 and PC2

Table S3. List of genes enriched in each group identified by principal-component analysis, related to Figure 3A

Table S4. List of genes included in each cluster (related to Figure S4C), the normalized read counts at each passage, and the biological process GO enrichment analysis results for each cluster (related to Figure S4D)

Table S5. List of genes significantly upregulated and downregulated in epithelial and mesenchymal enriched samples and GO (biological processes, molecular function, and cellular component) enrichment analysis

Document S2. Article plus supplemental information

## References

[bib1] Abu-Abed S., Dollé P., Metzger D., Beckett B., Chambon P., Petkovich M. (2001). The retinoic acid-metabolizing enzyme, CYP26A1, is essential for normal hindbrain patterning, vertebral identity, and development of posterior structures. Genes Dev..

[bib2] Aires R., de Lemos L., Novoa A., Jurberg A.D., Mascrez B., Duboule D., Mallo M. (2019). Tail bud progenitor activity relies on a network comprising Gdf11, Lin28, and Hox13 genes. Dev. Cell.

[bib3] Aires R., Jurberg A.D., Leal F., Nóvoa A., Cohn M.J., Mallo M. (2016). Oct4 is a key regulator of vertebrate trunk length diversity. Dev. Cell.

[bib4] Amin S., Neijts R., Simmini S., van Rooijen C., Tan S.C., Kester L., van Oudenaarden A., Creyghton M.P., Deschamps J. (2016). Cdx and T Brachyury Co-activate growth signaling in the embryonic axial progenitor niche. Cell Rep..

[bib5] Andersson O., Reissmann E., Ibanez C.F. (2006). Growth differentiation factor 11 signals through the transforming growth factor-beta receptor ALK5 to regionalize the anterior-posterior axis. EMBO Rep..

[bib6] Cano A., Perez-Moreno M.A., Rodrigo I., Locascio A., Blanco M.J., del Barrio M.G., Portillo F., Nieto M.A. (2000). The transcription factor snail controls epithelial-mesenchymal transitions by repressing E-cadherin expression. Nat. Cell Biol..

[bib7] Clovis Y.M., Seo S.Y., Kwon J.-s., Rhee J.C., Yeo S., Lee J.W., Lee S., Lee S.-K. (2016). Chx10 consolidates V2a interneuron identity through two distinct gene repression modes. Cell Rep..

[bib8] Cunningham T.J., Brade T., Sandell L.L., Lewandoski M., Trainor P.A., Colas A., Mercola M., Duester G. (2015). Retinoic acid activity in undifferentiated neural progenitors is sufficient to fulfill its role in restricting Fgf8 expression for somitogenesis. PLoS One.

[bib9] Cunningham T.J., Duester G. (2015). Mechanisms of retinoic acid signalling and its roles in organ and limb development. Nat. Rev. Mol. Cell Biol..

[bib10] Curran K., Lister J.A., Kunkel G.R., Prendergast A., Parichy D.M., Raible D.W. (2010). Interplay between Foxd3 and Mitf regulates cell fate plasticity in the zebrafish neural crest. Dev. Biol..

[bib11] Denans N., Iimura T., Pourquie O. (2015). Hox genes control vertebrate body elongation by collinear Wnt repression. Elife.

[bib12] Deschamps J., Duboule D. (2017). Embryonic timing, axial stem cells, chromatin dynamics, and the Hox clock. Genes Dev..

[bib13] Diez del Corral R., Breitkreuz D.N., Storey K.G. (2002). Onset of neuronal differentiation is regulated by paraxial mesoderm and requires attenuation of FGF signalling. Development.

[bib14] Diez del Corral R., Olivera-Martinez I., Goriely A., Gale E., Maden M., Storey K. (2003). Opposing FGF and retinoid pathways control ventral neural pattern, neuronal differentiation, and segmentation during body axis extension. Neuron.

[bib15] Francius C., Clotman F. (2014). Generating spinal motor neuron diversity: a long quest for neuronal identity. Cell. Mol. Life Sci..

[bib16] Frith T.J., Granata I., Wind M., Stout E., Thompson O., Neumann K., Stavish D., Heath P.R., Ortmann D., Hackland J.O. (2018). Human axial progenitors generate trunk neural crest cells in vitro. Elife.

[bib17] Gaunt S.J., George M., Paul Y.-L. (2013). Direct activation of a mouse Hoxd11 axial expression enhancer by Gdf11/Smad signalling. Dev. Biol..

[bib18] Gomez G.A., Prasad M.S., Sandhu N., Shelar P.B., Leung A.W., García-Castro M.I. (2019). Human neural crest induction by temporal modulation of WNT activation. Dev. Biol..

[bib19] Gomez G.A., Prasad M.S., Wong M., Charney R.M., Shelar P.B., Sandhu N., Hackland J.O.S., Hernandez J.C., Leung A.W., Garcia-Castro M.I. (2019). WNT/beta-catenin modulates the axial identity of embryonic stem cell-derived human neural crest. Development.

[bib20] Gouti M., Delile J., Stamataki D., Wymeersch F.J., Huang Y., Kleinjung J., Wilson V., Briscoe J. (2017). A gene regulatory network balances neural and mesoderm specification during vertebrate trunk development. Dev. Cell.

[bib21] Hackland J.O.S., Frith T.J.R., Thompson O., Marin Navarro A., Garcia-Castro M.I., Unger C., Andrews P.W. (2017). Top-down inhibition of BMP signaling enables robust induction of hPSCs into neural crest in fully defined, xeno-free conditions. Stem Cell Rep..

[bib22] Hackland J.O.S., Shelar P.B., Sandhu N., Prasad M.S., Charney R.M., Gomez G.A., Frith T.J.R., Garcia-Castro M.I. (2019). FGF modulates the axial identity of trunk hPSC-derived neural crest but not the cranial-trunk decision. Stem Cell Rep..

[bib23] Henrique D., Abranches E., Verrier L., Storey K.G. (2015). Neuromesodermal progenitors and the making of the spinal cord. Development.

[bib24] Janesick A., Nguyen T.T.L., Aisaki K.-i., Igarashi K., Kitajima S., Chandraratna R.A.S., Kanno J., Blumberg B. (2014). Active repression by RARγ signaling is required for vertebrate axial elongation. Development.

[bib25] Jessell T.M. (2000). Neuronal specification in the spinal cord: inductive signals and transcriptional codes. Nat. Rev. Genet..

[bib26] Jurberg A.D., Aires R., Varela-Lasheras I., Novoa A., Mallo M. (2013). Switching axial progenitors from producing trunk to tail tissues in vertebrate embryos. Dev. Cell.

[bib27] Kim K., Ossipova O., Sokol S.Y. (2015). Neural crest specification by inhibition of the ROCK/Myosin II pathway. Stem cells (Dayton, Ohio).

[bib28] Klein E.S., Pino M.E., Johnson A.T., Davies P.J., Nagpal S., Thacher S.M., Krasinski G., Chandraratna R.A. (1996). Identification and functional separation of retinoic acid receptor neutral antagonists and inverse agonists. J. Biol. Chem..

[bib29] Koch F., Scholze M., Wittler L., Schifferl D., Sudheer S., Grote P., Timmermann B., Macura K., Herrmann B.G. (2017). Antagonistic activities of Sox2 and Brachyury control the fate choice of neuro-mesodermal progenitors. Dev. Cell.

[bib30] Kumamaru H., Kadoya K., Adler A.F., Takashima Y., Graham L., Coppola G., Tuszynski M.H. (2018). Generation and post-injury integration of human spinal cord neural stem cells. Nat. Methods.

[bib31] Leung A.W., Murdoch B., Salem A.F., Prasad M.S., Gomez G.A., Garcia-Castro M.I. (2016). WNT/beta-catenin signaling mediates human neural crest induction via a pre-neural border intermediate. Development.

[bib32] Li X., Liu Z., Qiu M., Yang Z. (2014). Sp8 plays a supplementary role to Pax6 in establishing the pMN/p3 domain boundary in the spinal cord. Development.

[bib33] Lin Y.-M.J., Hsin I.L., Sun H.S., Lin S., Lai Y.-L., Chen H.-Y., Chen T.-Y., Chen Y.-P., Shen Y.-T., Wu H.-M. (2018). NTF3 is a novel target gene of the transcription factor POU3F2 and is required for neuronal differentiation. Mol. Neurobiol..

[bib34] Lippmann E.S., Williams C.E., Ruhl D.A., Estevez-Silva M.C., Chapman E.R., Coon J.J., Ashton R.S. (2015). Deterministic HOX patterning in human pluripotent stem cell-derived neuroectoderm. Stem Cel. Rep..

[bib35] Liu J.-P. (2006). The function of growth/differentiation factor 11 (Gdf11) in rostrocaudal patterning of the developing spinal cord. Development.

[bib36] Lukoseviciute M., Mayes S., Sauka-Spengler T. (2021). Neuromesodermal progenitor origin of trunk neural crest in vivo. bioRxiv.

[bib37] Luu B., Ellisor D., Zervas M. (2011). The lineage contribution and role of Gbx2 in spinal cord development. PLoS One.

[bib38] Martin B.L., Kimelman D. (2010). Brachyury establishes the embryonic mesodermal progenitor niche. Genes Dev..

[bib39] McGrew M.J., Sherman A., Lillico S.G., Ellard F.M., Radcliffe P.A., Gilhooley H.J., Mitrophanous K.A., Cambray N., Wilson V., Sang H. (2008). Localised axial progenitor cell populations in the avian tail bud are not committed to a posterior Hox identity. Development.

[bib40] McPherron A.C., Huynh T.V., Lee S.J. (2009). Redundancy of myostatin and growth/differentiation factor 11 function. BMC Dev. Biol..

[bib41] Mouilleau V., Vaslin C., Robert R., Gribaudo S., Nicolas N., Jarrige M., Terray A., Lesueur L., Mathis M.W., Croft G. (2021). Dynamic extrinsic pacing of the HOX clock in human axial progenitors controls motor neuron subtype specification. Development.

[bib42] Narboux-Neme N., Ekker M., Levi G., Heude E. (2019). Posterior axis formation requires Dlx5/Dlx6 expression at the neural plate border. PLoS One.

[bib43] Neijts R., Amin S., van Rooijen C., Deschamps J. (2017). Cdx is crucial for the timing mechanism driving colinear Hox activation and defines a trunk segment in the Hox cluster topology. Dev. Biol..

[bib44] Niederreither K., Subbarayan V., Dollé P., Chambon P. (1999). Embryonic retinoic acid synthesis is essential for early mouse post-implantation development. Nat. Genet..

[bib45] Olivera-Martinez I., Schurch N., Li R.A., Song J., Halley P.A., Das R.M., Burt D.W., Barton G.J., Storey K.G. (2014). Major transcriptome re-organisation and abrupt changes in signalling, cell cycle and chromatin regulation at neural differentiation in vivo. Development.

[bib46] Olivera-Martinez I., Storey K.G. (2007). Wnt signals provide a timing mechanism for the FGF-retinoid differentiation switch during vertebrate body axis extension. Development.

[bib47] Ribes V., Le Roux I., Rhinn M., Schuhbaur B., Dollé P. (2009). Early mouse caudal development relies on crosstalk between retinoic acid, Shh and Fgf signalling pathways. Development.

[bib48] Ribes V., Stutzmann F., Bianchetti L., Guillemot F., Dollé P., Le Roux I. (2008). Combinatorial signalling controls Neurogenin2 expression at the onset of spinal neurogenesis. Dev. Biol..

[bib49] Sakai Y., Meno C., Fujii H., Nishino J., Shiratori H., Saijoh Y., Rossant J., Hamada H. (2001). The retinoic acid-inactivating enzyme CYP26 is essential for establishing an uneven distribution of retinoic acid along the anterio-posterior axis within the mouse embryo. Genes Dev..

[bib50] Sandberg M., Kallstrom M., Muhr J. (2005). Sox21 promotes the progression of vertebrate neurogenesis. Nat. Neurosci..

[bib51] Sasai N., Kutejova E., Briscoe J. (2014). Integration of signals along orthogonal axes of the vertebrate neural tube controls progenitor competence and increases cell diversity. PLoS Biol..

[bib52] Shum A.S., Poon L.L., Tang W.W., Koide T., Chan B.W., Leung Y.C., Shiroishi T., Copp A.J. (1999). Retinoic acid induces down-regulation of Wnt-3a, apoptosis and diversion of tail bud cells to a neural fate in the mouse embryo. Mech. Dev..

[bib53] Storey K.G., Goriely A., Sargent C.M., Brown J.M., Burns H.D., Abud H.M., Heath J.K. (1998). Early posterior neural tissue is induced by FGF in the chick embryo. Development.

[bib54] Tsakiridis A., Huang Y., Blin G., Skylaki S., Wymeersch F., Osorno R., Economou C., Karagianni E., Zhao S., Lowell S., Wilson V. (2014). Distinct Wnt-driven primitive streak-like populations reflect in vivo lineage precursors. Development.

[bib55] van de Ven C., Bialecka M., Neijts R., Young T., Rowland J.E., Stringer E.J., Van Rooijen C., Meijlink F., Novoa A., Freund J.N. (2011). Concerted involvement of Cdx/Hox genes and Wnt signaling in morphogenesis of the caudal neural tube and cloacal derivatives from the posterior growth zone. Development.

[bib56] Verrier L., Davidson L., Gierlinski M., Dady A., Storey K.G. (2018). Neural differentiation, selection and transcriptomic profiling of human neuromesodermal progenitor-like cells in vitro. Development.

[bib57] Wind M., Gogolou A., Manipur I., Granata I., Butler L., Andrews P.W., Barbaric I., Ning K., Guarracino M.R., Placzek M., Tsakiridis A. (2021). Defining the signalling determinants of a posterior ventral spinal cord identity in human neuromesodermal progenitor derivatives. Development.

[bib58] Wymeersch F.J., Huang Y., Blin G., Cambray N., Wilkie R., Wong F.C., Wilson V. (2016). Position-dependent plasticity of distinct progenitor types in the primitive streak. Elife.

[bib59] Wymeersch F.J., Wilson V., Tsakiridis A. (2021). Understanding axial progenitor biology in vivo and in vitro. Development.

