# Supplementary material for: Rostrocaudal patterning and neural crest differentiation of human pre-neural spinal cord progenitors in vitro
Source: Stem Cell Reports. 2022 Mar 24;17(4):894–910. doi: 10.1016/j.stemcr.2022.02.018 (PMC9023813; doi:10.1016/j.stemcr.2022.02.018)
Supplement: Document S1. Supplemental experimental procedures, Figures S1–S7, and Tables S6 and S7 [file mmc1.pdf]

Stem Cell Reports, Volume *17*

Supplemental Information

Rostrocaudal patterning and neural crest differentiation of human pre-neural spinal cord progenitors *in vitro*

Fay Cooper, George E. Gentsch, Richard Mitter, Camille Bouissou, Lyn E. Healy, Ana Hernandez Rodriguez, James C. Smith, and Andreia S. Bernardo

SUPPLEMENTAL FIGURES

Figure S1

A

Paper	CHIR 99021	FGF	Time after treatment	Plating density	Recovery time after plating	Additional molecules
Frith et al., 2018	3 - 4 μM	FGF2 (20 ng/ml)	72h	55,000 cells/cm^2	0h	
Gouti et al., 2014	3 μM	FGF2 (20 ng/ml)	72h	80,000 cells/cm^2	0h	
Verrier et al., 2018	3 μM	FGF2 (20 ng/ml)	72h	40,000 cells/cm^2	24h	48h-72h: SB-431542 (10 μM), Noggin (50ng/ml)
Lippman et al., 2016	2-3 μM	FGF8b (200 ng/ml)	72h (FGF 24h, FGF + CHIR 48h)	100,000 cells/cm^2	24h	
Denham et al., 2015	3 μM	None	96h	Aggregates	0h	SB-431542 (10 μM)
Wang et al., 2019	10 μM	FGF2 (20 ng/ml)	48h	200,000 cells/cm^2	0h	TGFβ1 (2-5 ng/ml)
Kumamaru et al., 2018	4 μM	FGF2 (100 ng/ml) or FGF8b (100 ng/ml)	72h	70% confluence	N/A	LDN-193189 (100 nM), SB-431542 (10 μM), DAPT (10 μM)
Gomez et al., 2019	7 - 12 μM	None	24h - 48h	200,00 cells/cm^2	0h	
Hackland et all (2019)	3 μM	None	48h	20,000 cells/cm^2	0h	

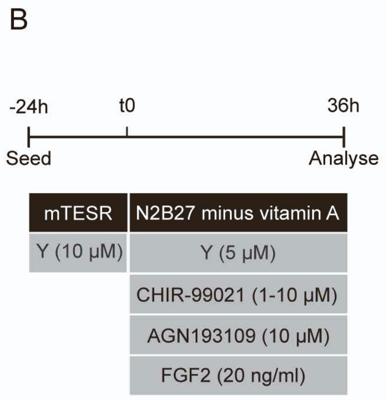

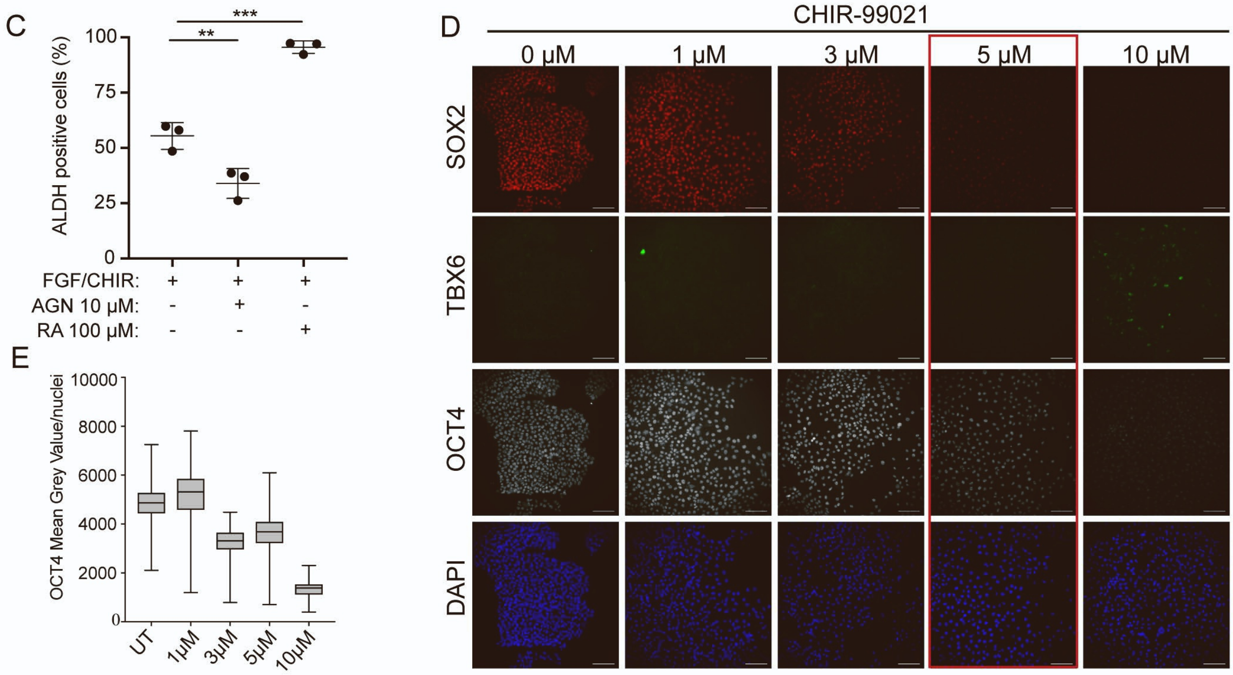

Figure S2

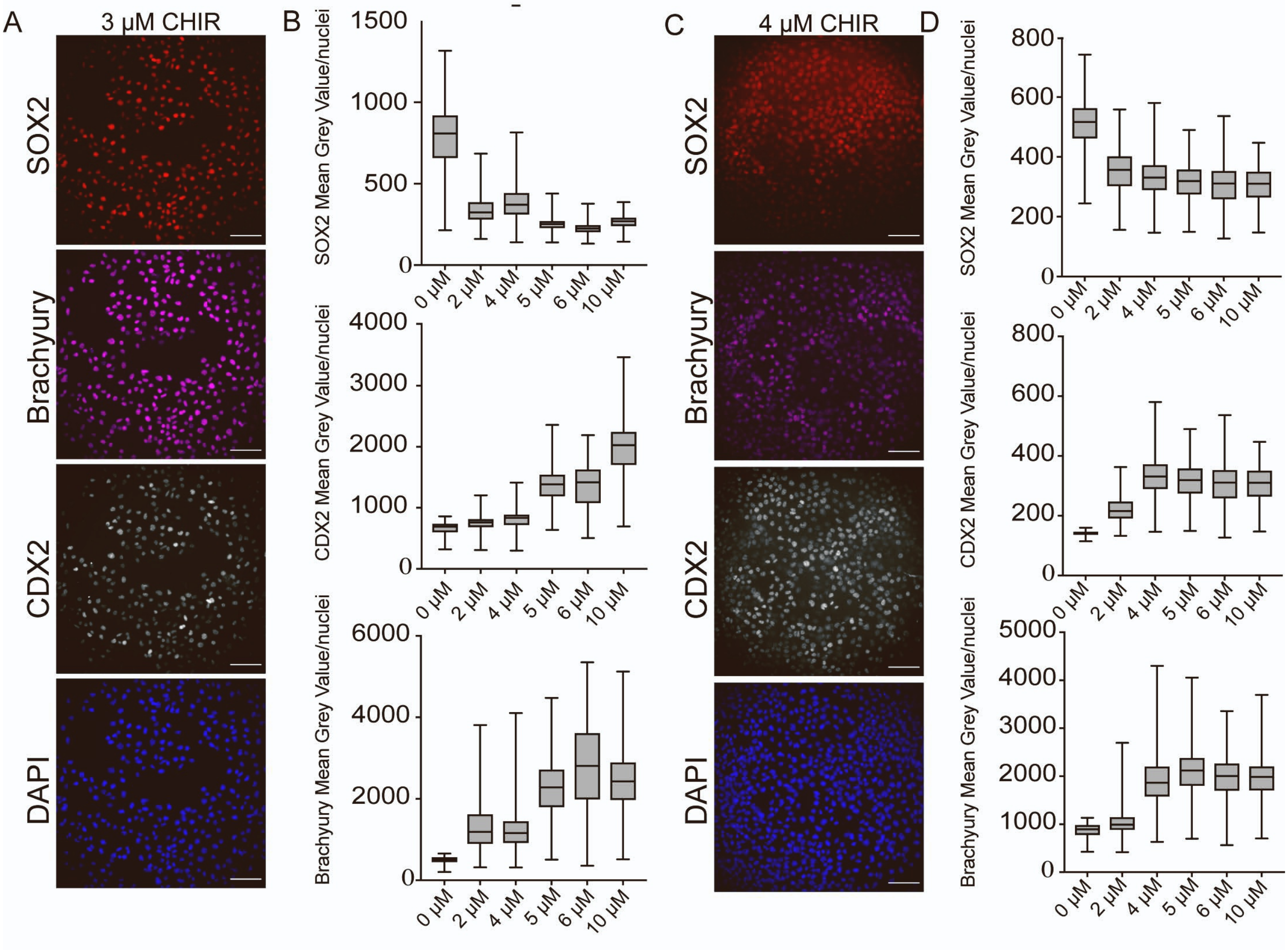
A
3 µM CHIR
SOX2
Brachyury
CDX2
DAPI
B
SOX2 Mean Grey Value/nuclei
CDX2 Mean Grey Value/nuclei
Brachyury Mean Grey Value/nuclei
0 µM
2 µM
4 µM
5 µM
6 µM
10 µM
C
4 µM CHIR
D

Figure S3

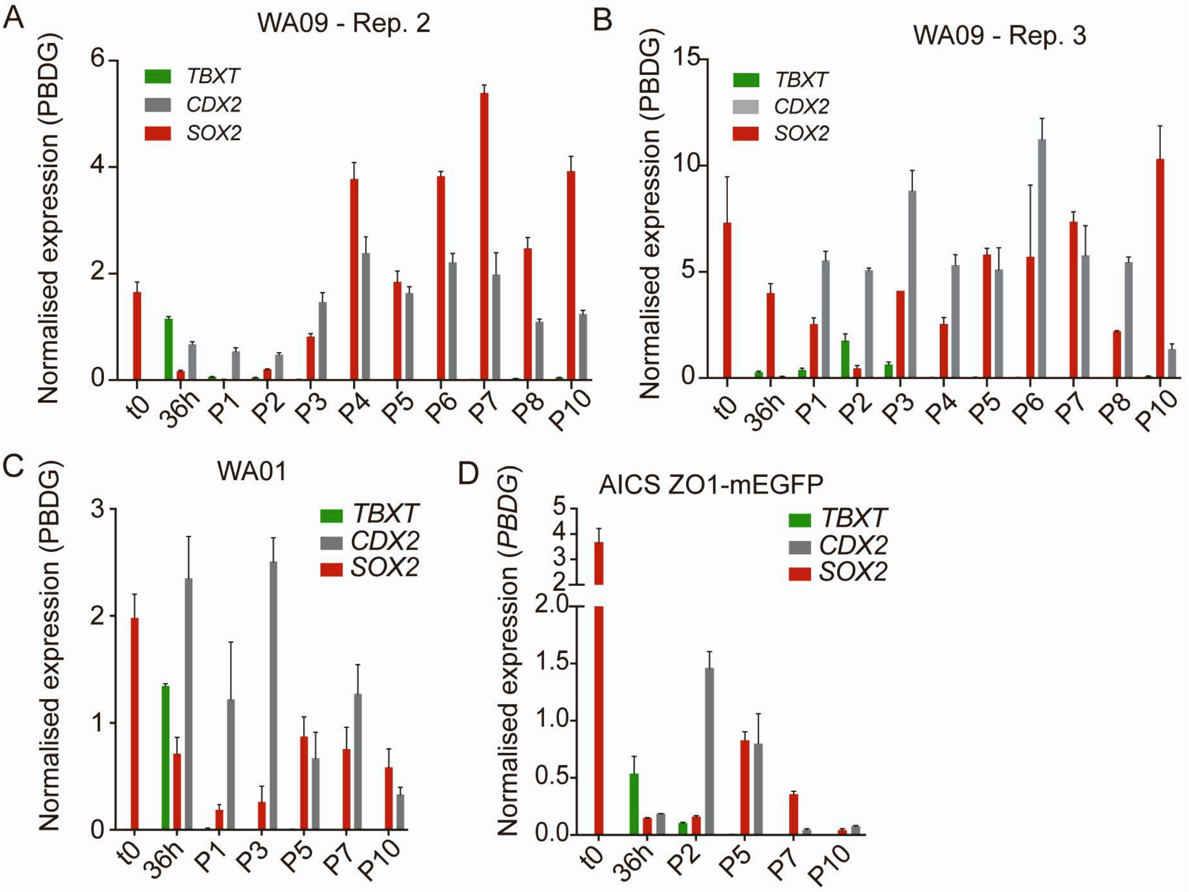
A
WA09 - Rep. 2
B
WA09 - Rep. 3
C
WA01
D
AICS ZO1-mEGFP
TBXT
CDX2
SOX2
Normalised expression (PBDG)
t0
36h
P1
P2
P3
P4
P5
P6
P7
P8
P10

Figure S4

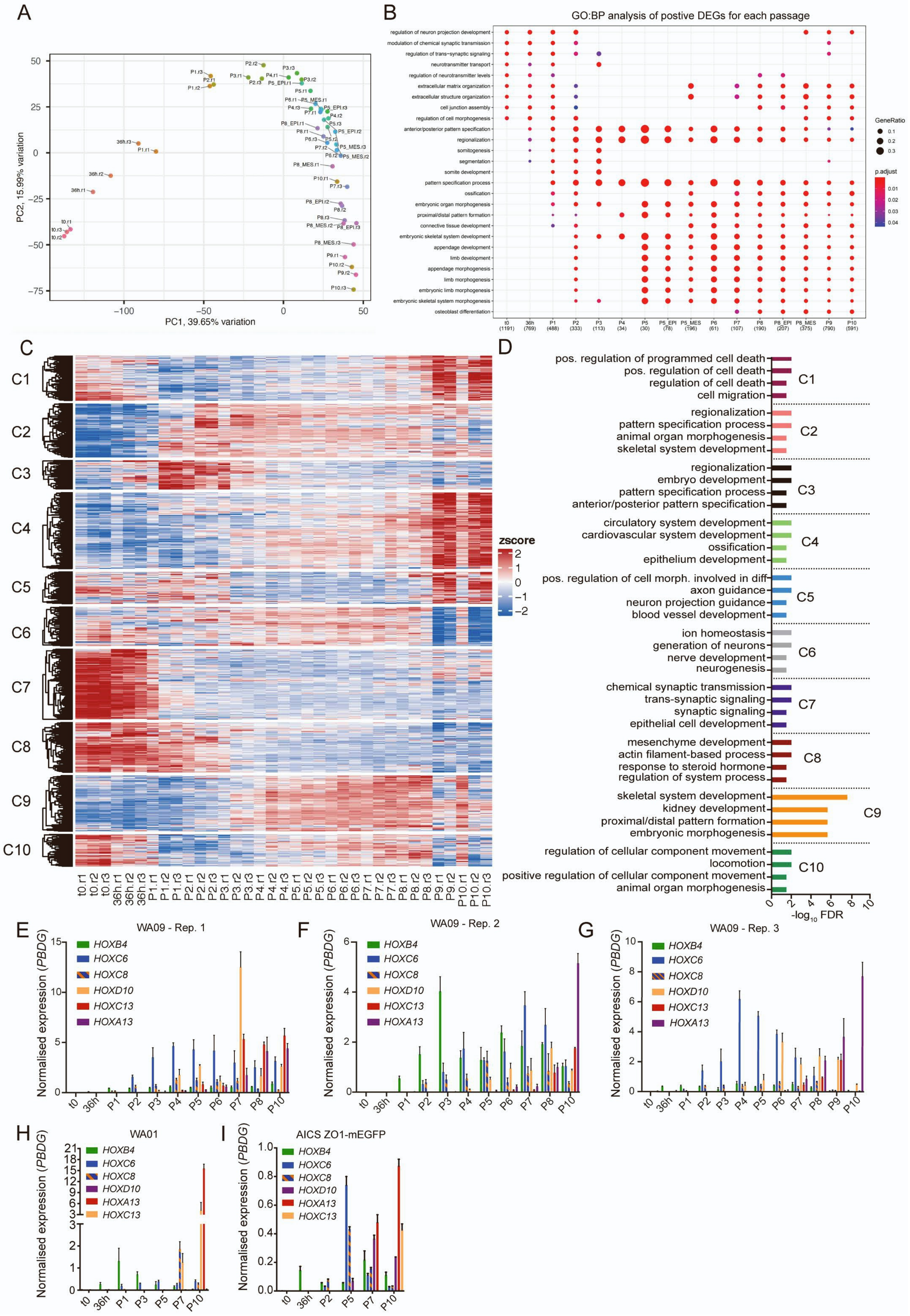
A
PC1, 39.65% variation
PC2, 15.99% variation
B
GO:BP analysis of postive DEGs for each passage
regulation of neuron projection development
modulation of chemical synaptic transmission
regulation of trans−synaptic signaling
neurotransmitter transport
regulation of neurotransmitter levels
extracellular matrix organization
extracellular structure organization
cell junction assembly
regulation of cell morphogenesis
anterior/posterior pattern specification
regionalization
somitogenesis
segmentation
somite development
pattern specification process
ossification
embryonic organ morphogenesis
proximal/distal pattern formation
connective tissue development
embryonic skeletal system development
appendage development
limb development
appendage morphogenesis
limb morphogenesis
embryonic limb morphogenesis
embryonic skeletal system morphogenesis
osteoblast differentiation
GeneRatio
p.adjust
C
zscore
D
pos. regulation of programmed cell death
pos. regulation of cell death
regulation of cell death
cell migration
regionalization
pattern specification process
animal organ morphogenesis
skeletal system development
embryo development
anterior/posterior pattern specification
circulatory system development
cardiovascular system development
ossification
epithelium development
pos. regulation of cell morph. involved in diff
axon guidance
neuron projection guidance
blood vessel development
ion homeostasis
generation of neurons
nerve development
neurogenesis
chemical synaptic transmission
trans-synaptic signaling
synaptic signaling
epithelial cell development
mesenchyme development
actin filament-based process
response to steroid hormone
regulation of system process
skeletal system development
kidney development
proximal/distal pattern formation
embryonic morphogenesis
regulation of cellular component movement
locomotion
positive regulation of cellular component movement
animal organ morphogenesis
-log10 FDR
E
WA09 - Rep. 1
F
WA09 - Rep. 2
G
WA09 - Rep. 3
H
WA01
I
AICS ZO1-mEGFP
Normalised expression (PBDG)
HOXB4
HOXC6
HOXC8
HOXD10
HOXC13
HOXA13

Figure S5

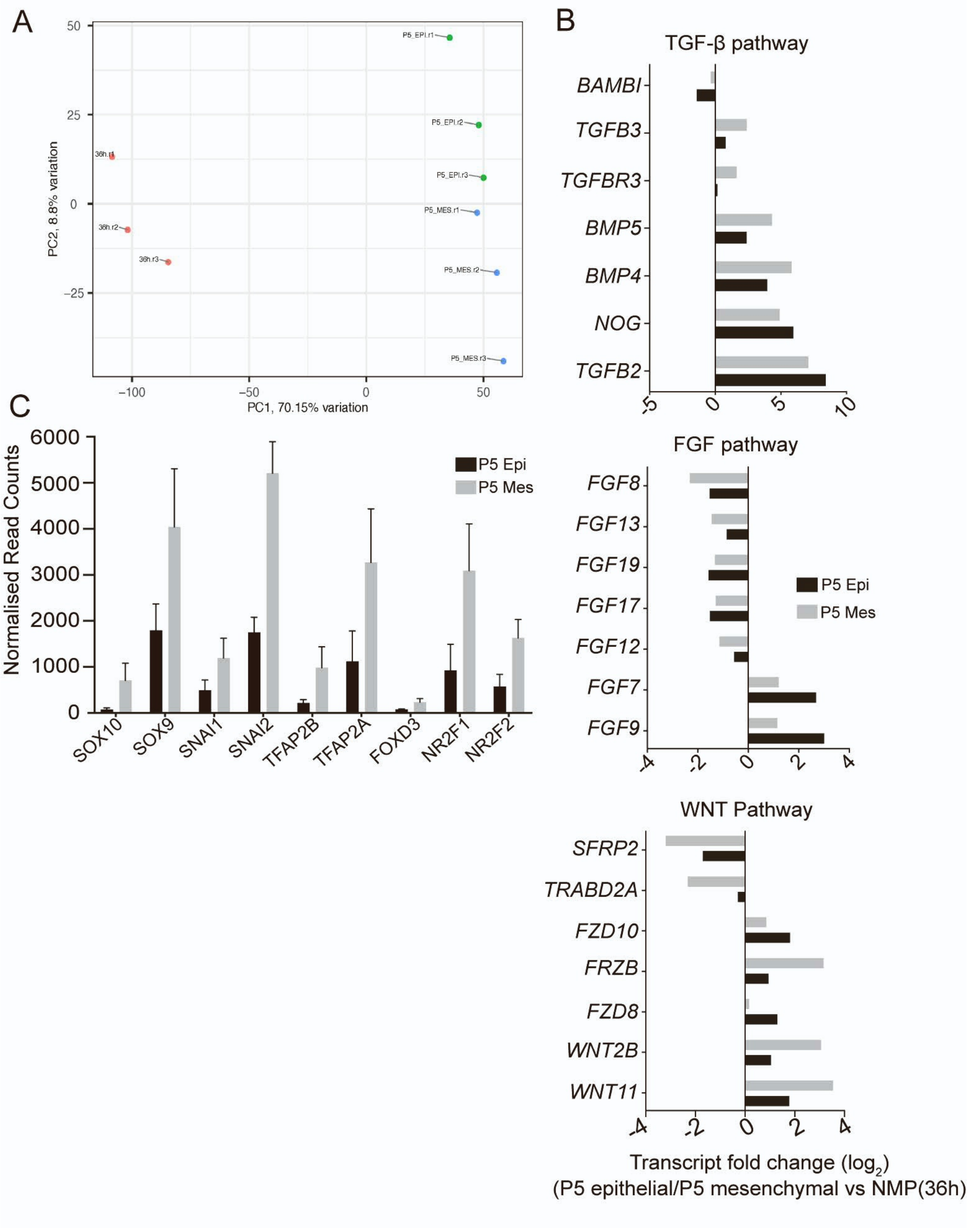

A
B
C
PC1, 70.15% variation
PC2, 8.8% variation
TGF-β pathway
BAMBI
TGFB3
TGFBR3
BMP5
BMP4
NOG
TGFB2
FGF pathway
FGF8
FGF13
FGF19
FGF17
FGF12
FGF7
FGF9
P5 Epi
P5 Mes
WNT Pathway
SFRP2
TRABD2A
FZD10
FRZB
FZD8
WNT2B
WNT11
Normalised Read Counts
SOX10
SOX9
SNAI1
SNAI2
TFAP2B
TFAP2A
FOXD3
NR2F1
NR2F2
Transcript fold change (log2)
(P5 epithelial/P5 mesenchymal vs NMP(36h)

Figure S6

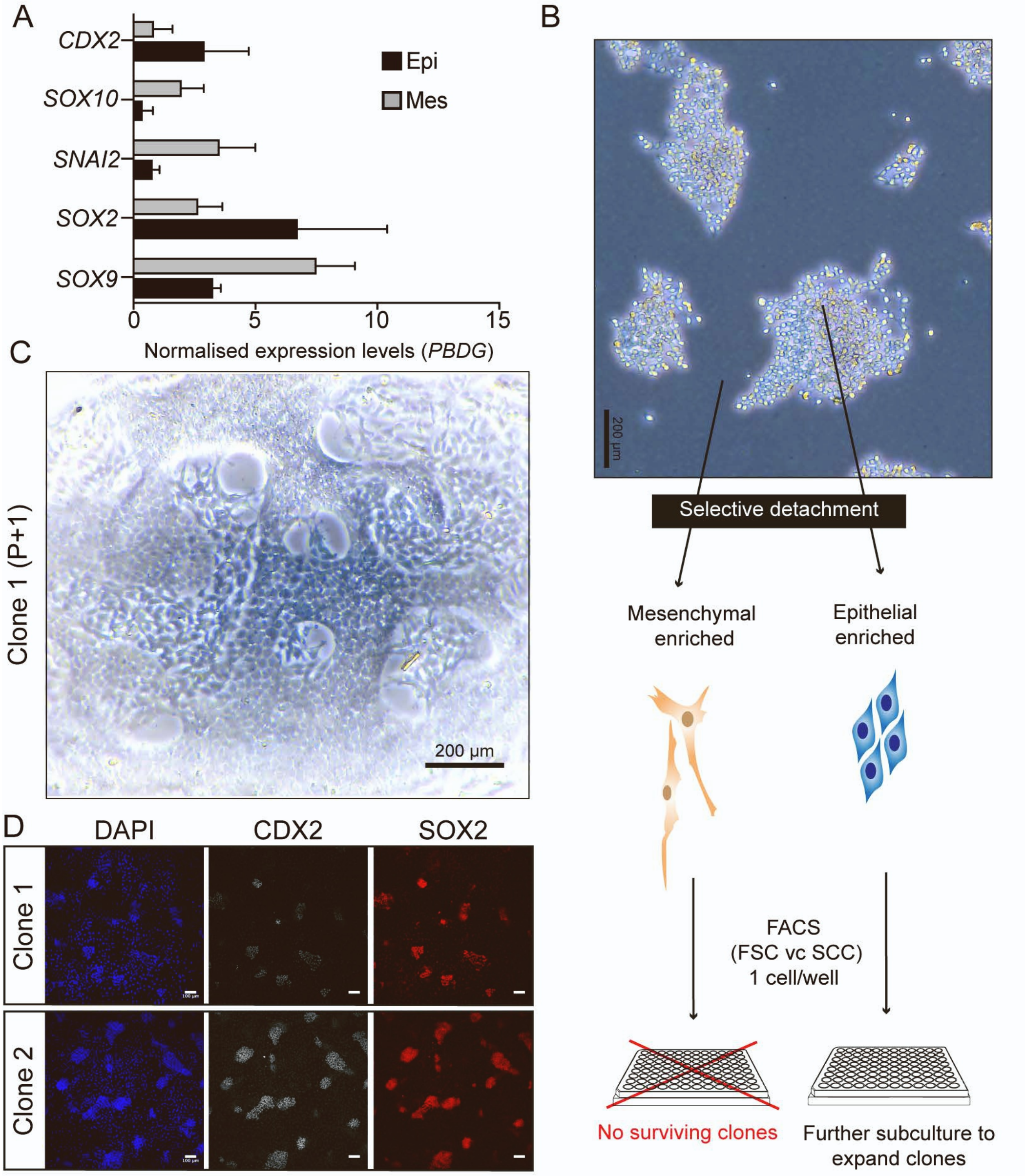

A
CDX2
SOX10
SNAI2
SOX2
SOX9
Epi
Mes
0
5
10
15
Normalised expression levels (PBDG)
B
200 µm
Selective detachment
Mesenchymal enriched
Epithelial enriched
FACS (FSC vc SCC) 1 cell/well
No surviving clones
Further subculture to expand clones
C
Clone 1 (P+1)
200 µm
D
DAPI
CDX2
SOX2
Clone 1
Clone 2

Figure S7

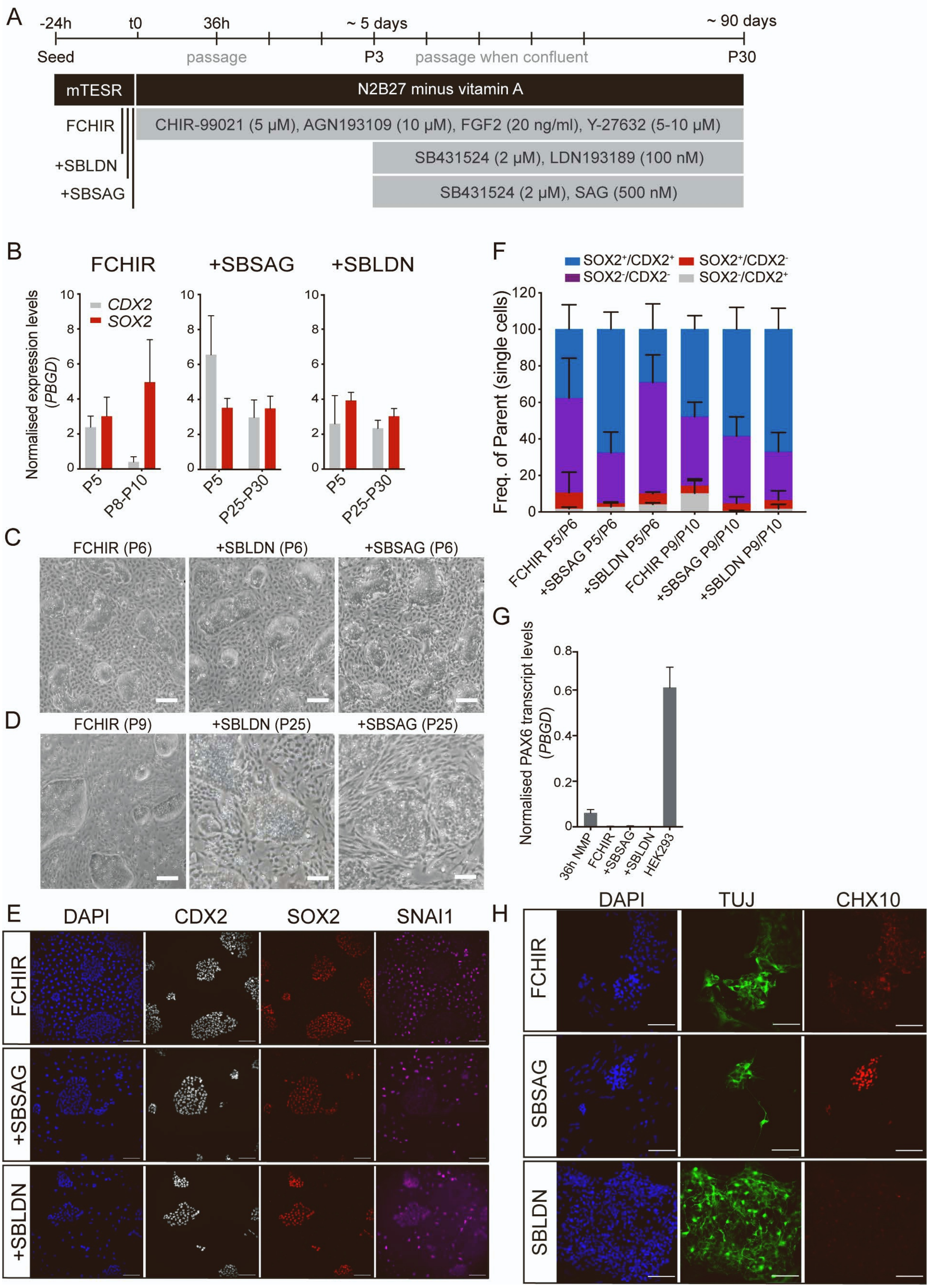

A
-24h
t0
36h
~ 5 days
~ 90 days
Seed
passage
P3
passage when confluent
P30
mTESR
N2B27 minus vitamin A
FCHIR
CHIR-99021 (5 μM), AGN193109 (10 μM), FGF2 (20 ng/ml), Y-27632 (5-10 μM)
+SBLDN
SB431524 (2 μM), LDN193189 (100 nM)
+SBSAG
SB431524 (2 μM), SAG (500 nM)
B
FCHIR
+SBSAG
+SBLDN
Normalised expression levels (PBGD)
CDX2
SOX2
P5
P8-P10
P25-P30
F
SOX2+/CDX2+
SOX2+/CDX2-
SOX2-/CDX2-
SOX2-/CDX2+
Freq. of Parent (single cells)
FCHIR P5/P6
+SBSAG P5/P6
+SBLDN P5/P6
FCHIR P9/P10
+SBSAG P9/P10
+SBLDN P9/P10
C
FCHIR (P6)
+SBLDN (P6)
+SBSAG (P6)
D
FCHIR (P9)
+SBLDN (P25)
+SBSAG (P25)
G
Normalised PAX6 transcript levels (PBGD)
36h NMP
FCHIR
+SBSAG
+SBLDN
HEK293
E
DAPI
CDX2
SOX2
SNAI1
FCHIR
+SBSAG
+SBLDN
H
DAPI
TUJ
CHX10
FCHIR
SBSAG
SBLDN

SUPPLEMENTAL FIGURE LEGENDS

Figure S1: NMP-like cells are induced by combined Wnt/FGF and inhibited RA signalling, related to Figure 1.
A) Summary of protocols used in recent studies to generate NMP-like cells from hPSCs. Table includes plating density and recovery time after plating, as well as the exogenous molecules and treatment time used (Wang et al., 2019, Gouti et al., 2014, Frith et al., 2018, Edri et al., 2019, Verrier et al., 2018, Lippmann et al., 2015, Gomez et al., 2019, Kumamaru et al., 2018, Denham et al., 2015, Hackland et al., 2019). B) Tissue culture scheme for optimising NMP generation from hPSCs. C) ALDEFLUOR assay was used to measure the expression of aldehyde dehydrogenases (ALDH) in 36h samples generated in three conditions: 1) FGF2 and CHIR only, 2) FGF, CHIR and AGN or 3) FGF, CHIR and RA. Samples were analysed using flow cytometry and results were presented as the percentage of cells expressing ALDH. Error bars show SD (n = 3 independent replicates). **P <0.01, ***P <0.001 (ANOVA). D) Representative immunostaining SOX2 (red), TBX6 (green) OCT4 (grey) and the nuclear stain DAPI (blue) after 36h treatment following scheme as shown in Figure 1A with 0 µM, 1 µM, 3 µM, 5 µM and 10 µM CHIR-99021. Scale bars, 100 µm. E) Box-plot showing mean grey value/nuclei quantified from repeat experiments as shown in (D). Plot show data points collected from 2 experiments (>450 nuclei/experiment).

Figure S2: Generation of NMP-like cells in multiple hPSC lines requires modulation of the Wnt pathway, relating to Figure 1
A, B) Optimal CHIR concentration (3 µM) was optimised in WA01 (H1) hESCs. (A) Representative immunostaining of NMP markers SOX2 (red) and CDX2 (grey) and Brachyury (magenta) at 36h after following treatment scheme with 3 µM CHIR and (B) quantification markers over a range of CHIR concentrations between 1-10 µM. Scale bars, 100µm. C, D) Optimal CHIR concentration (4 µM) was optimised in AICS ZO1-mEGFP (AICS-0024) iPSCs hESCs. (C) Representative immunostaining of NMP markers SOX2 (red) and CDX2 (grey) and Brachyury (magenta) at 36h after following treatment scheme with 3 µM CHIR and (D) quantification markers over a range of CHIR concentrations between 1-10 µM. Scale bars, 100µm.

Figure S3: CDX2 and SOX2 expression can be maintained for 10 passages, relating to Figure 2.
A,B) Transcriptional analysis (RT-qPCR) of two independent experiments showing NMP markers *TBXT, SOX2* and *CDX2* at each passage, up to passage 10. Expression levels are normalised to the reference gene *PBDG*. Error bars show SD, (n = 3 technical replicates). C,D) Transcriptional analysis (RT-qPCR) of NMP markers *TBXT, SOX2* and *CDX2* in either WA09 or AICS ZO1-mEGFP up to passage 10. Expression levels are normalised to the reference gene *PBDG*. Error bars show SD, (n = 3 technical replicates).

Figure S4: Principal component, hierarchical clustering and HOX gene expression analysis samples collected over passaging, relating to Figure 3.
A) PCA depicting variance between timepoints (t0-P10) and replicates analysed by RNA-seq. B) Dotplot showing the top 50 protein coding GO:BP terms associated with the top 50 protein coding genes significantly enriched at each passage compared to t0. C) Heatmap showing dynamically expressed genes (z-score) sorted into 10 clusters (C1-10) using k-means hierarchical clustering. Each cluster represents a different temporal expression pattern. D) Biological processes GO analysis for gene sets in each cluster shown in (C). E,F,G) Transcriptional analysis (RT-qPCR) of three independent experiments showing selected *HOX* genes at each passage up to passage 10. Expression levels are normalised to the reference gene *PBDG*. Error bars show SD, (n = 3 technical replicates). H,I) Transcriptional analysis (RT-qPCR) selected *HOX* genes in either WA09 or AICS ZO1-mEGFP up to passage 10. Expression levels are normalised to the reference gene *PBDG*. Error bars show SD, (n = 3 technical replicates).

Figure S5: Principal component analysis of mesenchymal and epithelial samples analysed by bulk RNA-sequencing, relating to Figure 4.
A) PCA analysis showing biological replicates for the mesenchymal (MES) and epithelial (EPI) enriched samples and NMP samples (36h). B) Graphs showing transcriptional fold change (FC) of selected TGF-β superfamily genes (top), FGF signalling genes (middle) and WNT (bottom) in P5 epithelial and P5 mesenchymal samples over 36h samples. All genes shown are significantly changed between P5 MES and EPI samples (FDR <1%, a fold change of at least ± 2, and a base mean >100). C) Normalised expression levels of known markers of NC genes (*SOX10, SOX9, SNAI1, SNAI2, TFAP2B, TFAP2A,*

FOXD3, NR2F1 and *NR2F2*) which are significantly upregulated in mesenchymal enriched samples compared to epithelial as determined by RNA-seq. Error bars show SEM (n = 3 independent replicates).

Figure S6: Generating sub-clonal populations from PNP/NC cell enriched samples, relating to Figure 5.
A) Normalised expression levels of *CDX2, SOX10, SNAI2, SOX2, SOX9* in mesenchymal and epithelial cells after enrichment and serial passaging for four passages (P+4). Error bars show SEM (n = 2 independent enrichment/passaging experiments). B) Scheme to generate sub-clonal populations from mesenchymal- or epithelial- enriched samples. Cells were selectively detached to separate epithelial from mesenchymal cell populations and single cells from each enriched cell sample were sorted (FACS) into wells of a 96 well plate. Surviving sub-clones were expanded for analysis. C) Representative bright-field image of a sub-clone generated from the epithelial enriched fragment after 1 passage. Scale bar, 200 µm. D) Representative immunostaining analysis of CDX2 (grey), SOX2 (red) and nuclear stain DAPI (blue) in two independent sub-clones generated from the epithelial enriched samples after 4 serial passages. Scale bar, 100 µm.

Figure S7: Further characterisation of +SBSAG and +SBLDN PNPs and downstream neurons, relating to Figure 6 and 7.
A) Scheme for generating and maintaining PNPs. B) Transcriptional quantification (RT-qPCR) of *CDX2* and *SOX2* at early (P5) and in late passage PNPs. Expression levels normalised to the reference gene *PBGD.* Error bars show SEM (n = 3-5 independent differentiations). C, D) Representative brightfield images and PNPs/NC at mid (P5) and late passages (FCHIR:P10, +SBLDN and +SBSAG: P25). Scale bar, 200 µm. E) Representative immunostaining of P5 cells for CDX2 (grey), SOX2 (red) and SNAI1 (magenta). Scale bar, 100 µM. F) SOX2/CDX2 flow cytometry analysis of FCHIR and +SBLDN and +SBSAG samples at early and late passages. Cells were analysed using SOX2 and CDX2 conjugated antibodies and plotted as percentage of expression. Error bars show mean with SEM (n = 3 independent differentiations). G) Quantification of *PAX6* transcript levels under various conditions as indicated in A, and in comparison to HEK293 (positive control) cells. Expression levels were normalised to reference gene *PBGD.* Error bars show mean with SEM (n = 2-3 independent differentiations). H) Representative immunostaining of ventral neurons stained with CHX10 (red) paired with βIII-tubulin (TUJ, green) and nuclear stain DAPI (blue). Scale bars, 100µm.

SUPPLEMENTAL TABLES

Table S1: List of genes significantly up- and down- regulated at 36h (NMP) compared to undifferentiated hESCs including fold change and FDR values. This information is located in the attached spreadsheet.

Table S2: List of top loading genes for PC1 and PC2. This information is located in the attached spreadsheet.

Table S3: List of genes enriched in each group identified by principal component analysis (relating to Figure 3A). This information is located in the attached spreadsheet.

Table S4: List of genes included in each cluster (relating to Figure S4C), the normalised read counts at each passage and the biological process GO enrichment analysis results for each cluster (relating to Figure S4D). This information is located in the attached spreadsheet.

Table S5: List of genes significantly up- and down- regulated in epithelial and mesenchymal enriched samples and GO (biological processes, molecular function and cellular component) enrichment analysis. This information is located in the attached spreadsheet.

Table S6: List of antibodies and conditions used for immunolabelling assays.

Gene name	Company	Reference	Dilution
SOX2	Millipore	AB5603	1/200
CDX2	Abcam	ab157524	1/150
SNAI1	R&D	AF3639	1/100
PAX6	Biolegend	Poly19013	1/100
SOX10	Cell signalling	89356	1/800
ETS1	Cell signalling	14069	1/1000
Brachyury	R&D	AF2085	1/200
TUJ	R&D	MAB1195	1/5000
TUJ	Biolegend	802001	1/5000
CHX10	Santa Cruz	sc-365519	1/100
NEUN	Sigma Aldrich	MAB377	1/100
ISL1	R&D	AF1837	1/200
TBX6	R&D	AF4744	1/100
OCT4	Santa Cruz	sc-5279	1/150
NANOG	R&D	AF1997	1/200
SOX9	Cell signalling	D8G8H	1/400
LHX1	Abcam	ab14554	1/500
LHX3	Abcam	ab14555	1/500
FOXP1	R&D	AF4634	1/500

Table S7: List of qRTPCR primers**.**

Gene name	Forward primer (5′-3′)	Reverse primer (5′-3′)
HOXB4	GCAAAGAGCCCGTCGTCTAC	GTCAGGTAGCGGTTGTAGTGAAAT
HOXA9	CAGGGTCTGGTGTTTTGTATAGGG	ACGCTTGACACTCACACTTTGTCC
HOXC6	CCAGGACCAGAAAGCCAGTA	TGGTACCGCGAGTAGATCTG
HOXC8	AGGAACCTGATGGAAACCTGAAGG	ATCAAACAGCGAAGGAGAGGAAGG
HOXD10	AAGGAAAGCAAAGAGGAAATCAA	TCGCGGGTGAGGTACATATT
HOXA13	GCCAAATGTACTGCCCCAAA	CCTTGGTATAAGGCACGCG
HOXC13	TCTCCCTTCCCAGACGTG	CCGGCGCTTCTCTTTGGT
PAX6	CGAGATTTCAGAGCCCCATA	AAGACACCACCGAGCTGATT
SOX2	TGGACAGTTACGCGCACAT	CGAGTAGGACATGCTGTAGGT
CDX2	TGCGAGTGGATGCGGAAG	AACTCCTTCTCCAGCTCCAG
TBXT	TGCTTCCCTGAGACCCAGTT	GATCACTTCTTTCCTTTGCATCAAG
PBGD	ATTACCCCGGGAGACTGAAC	GGCTGTTGCTTGGACTTCTC
SOX9	AACGCCGAGCTCAGCAAG	GTGGTCCTTCTTGTGCTGC
GDF11	AACGCCTTTGATCCCAGTGG	TGTTCTCTAGGACTCGAAGCTC
LIN28A	GCGCAGATCAAAAGGAGACA	GCGGACATGAGGCTACCATA
SOX10	CCCACTTGGACCACCGGCAC	TGGTTGGAGGGGTGGGTGGG
SNAI2	TGGTTGTGGTATGACAGGCA	AGCATTTCAACGCCTCCAAA

SUPPLEMENTAL EXPERIMENTAL PROCEDURES

Human pluripotent stem cell culture

All experiments were completed within 15 passages from cryopreservation and screened for mycoplasma monthly. Prior to cryopreservation, hPSCs were assessed for genetic stability by KaryoStat and indicators of pluripotency were assessed by PluriTest (Thermo Fisher Scientific). hPSCs were subject to routine pluripotency assays using BD Stemflow Human and Mouse Pluripotent Stem Cell Analysis Kit (BD Biosciences, 560477) as recommended by the manufacturers, or by immunostaining against OCT3/4, SOX2 and NANOG (see Table S6 for antibody details) using the standard immunostaining protocol below.

NMP differentiation

Human ESCs or iPSCs were dissociated into single cells using Gibco TrypLE Express (Thermo Fisher Scientific, 12604013) and plated at a density of 50,000 cells/cm^2 on Matrigel hESC-Qualified Matrix (Corning Incorporated, 354277). Cells were plated in mTESR1 supplemented with 10 μM Y-27632 (Tocris, 1254) for a 24h to 36h to allow recovery before starting differentiation into NMPs. Following recovery time, cells were grown in Dulbecco's Modified Eagle Medium/Nutrient Mixture F-12 (DMEM/F-12, Thermo Fisher Scientific, 10565018) supplemented with 1x Gibco B-27 supplement minus vitamin A (Thermo Fisher Scientific, 12587010) and 1x Gibco N2 (Thermo Fisher Scientific, 17502048), 4-6 μM CHIR-99021 (Selleck Chem, S2924-SEL-5mg), 10 μM AGN193109 sodium salt (Santa Cruz, sc-210768) and 20 ng/ml FGF2 (R&D systems, 233-FB-025) referred to from now on as NMP differentiation medium. NMP differentiation medium was supplemented with and 5 μM Y-27632 (Tocris).

PNP long term culture

During passage 1 to 3 progenitors were found to detach from the dish forming spheres. If this occurred, spheres were dissociated into single cells and re-plated immediately. PNP generation was more successful if cells did not detach, therefore, to prevent cells detaching during this period cells were passaged before reaching high confluency. In addition, cells were only removed from the 37°C incubator when ready to passage, as the temperature fluctuations promoted detachment. From passage 3 cells were grown in NMP differentiation medium supplemented with 5 μM Y-27632 (Tocris). Human iPSCs were found to detach more readily than hESCs. PNPs could be maintained, for 8 to 12 passages using standard conditions as above, passaging every 3-4 days when 80-90% confluent. To lock A-P axis progression, 2 μM SB431542 (CELL guidance systems, SM33-10) and 100 nM LDN193189 (Sigma-Aldrich, SML0559-5MG) or SB431542 (CELL guidance systems, SM33-10) and 500 nM smoothened agonist (SAG, Sigma-Aldrich, 566660-1mg) were added to NMP differentiation medium at passage 3 (Cuny et al., 2008, Halder et al., 2005, Inman et al., 2002). For selective detachment, 90% confluent PNPs were washed with PBS and treated with TrypLE express (Thermo Fisher Scientific) at 37°C for 3-5 mins. When mesenchymal cells started to detach, cells were gently removed by tilting the plate side-to-side. TrypLE containing the detached mesenchymal cells was carefully removed. Remaining epithelial cells were washed off the vessel using basal medium. For sacral HOX gene induction, cultures between P25 and P30 were split into long-term PNP maintenance medium (+SBSAG/+SBLDN), NMP differentiation medium (-SBSAG/-SBLDN) or NMP differentiation medium supplemented with 50 ng/ml GDF11 (Peprotech, 120-11-B). Samples were collected for RNA analysis when confluent (48-72h).

Clonal expansion of PNPs and NC cells

Passage 5 cells were selectively detached and dissociated into single cells using TrypLE express (Thermo Fisher Scientific) as described in supplemental experimental procedures. Cells were resuspended into RPMI 1640 (Thermo Fisher Scientific, 32404-014) supplemented with 10% (v/v) KnockOut serum replacement, (KSR, Thermo Fisher Scientific, 10828028) and 10 μM Y-27632 (Tocris*).* Cells were sorted using a MoFlo XPD (Beckman Coulter) using FSC and SSC profile to select single cells. Cells were sorted into Matrigel hESC-Qualified Matrix (Corning*)* coated 96 well plates containing NMP differentiation medium*.* Surviving cells were subsequently passaged using TrypLE express (Thermo Fisher Scientific).

Neuronal differentiation

80-90% confluent PNP/NC cultures were dissociated to single cells and plated at 33,000 cells/cm^2 onto Matrigel hESC-Qualified matrix (Corning) into the applicable former culture medium (NMP differentiation medium plus or minus SBLDN or SBSAG). 24h after plating, medium was replaced with neural differentiation medium consisting of Gibco neural basal medium (Thermo Fisher Scientific, 21103049) supplemented with Gibco 1x B27 supplement (Thermo Fisher Scientific, 17504044) and 1x

N2 (Thermo Fisher Scientific), 2 μM DAPT (Chem Cruz, sc-201315) and 1 μM retinoic acid (RA, Sigma Aldrich, sc-210768) for 48h. Following 48h treatment, media was replaced with 10 ng/ml brain-derived neurotrophic factor (BDNF, PeproTech, 450-02-2UG), 10 ng/ml glial-derived neurotrophic factor (GDNF, PeproTech, 450-10-2UG), 1 μM retinoic acid (RA, Sigma Aldrich, sc-210768), 1 μM cAMP (Sigma Aldrich, A6885-100mg) and 200 μM L-ascorbic acid (Sigma Aldrich, A8960) for 10 days. At day 12 cells were dissociated using TrypLE express and replated as single cells onto fresh Matrigel hESC-Qualified matrix (Corning) plates into neural differentiation medium (as above) supplemented with 20 μM DAPT (Chem Cruz, sc-201315), 10 ng/ml brain-derived neurotrophic factor (BDNF, PeproTech, 450-02-2UG), 10 ng/ml glial-derived neurotrophic factor (GDNF, PeproTech, 450-10-2UG), 1 μM cAMP (Sigma Aldrich, A6885-100mg) and 200 μM L-ascorbic acid (Sigma Aldrich, A8960). Medium was supplemented with 10 μM Y-27632 (Tocris) for the first 24h. During neural induction and maintenance, growth medium was replaced every 48h until day 24.

Immunofluorescence microscopy

Cells were cultured in 8 or 12 well μ-slides (Ibidi) and fixed with ice-cold 4% Pierce formaldehyde (w/v) methanol-free (Thermo Fisher Scientific, 28908) in PBS for 10-15 mins. Cells were permeabilised in PBS supplemented with 0.1 % (v/v) Triton-X100 (Sigma Aldrich, T8787-250ML) for 10 mins and blocked using PBS supplemented with 0.1 % (v/v) Triton-X100 (Sigma Aldrich), 5% (v/v) Donkey serum (Merck Millipore, S30-100ML). Primary antibodies were incubated in blocking solution at 4°C overnight (detailed in Table S6). Cells were then washed in PBS and incubated in Donkey AlexaFluor conjugated secondary antibodies (Abcam) diluted at 1:400 in blocking solution. Cells were mounted in Vectorshield antifade mounting medium containing DAPI (Vector Laboratories, H-1200-10). Cells were imaged using two imaging systems; 1) by a Zeiss LSM710 confocal microscope (Carl Ziess AG) using Zeiss Plan-Apochromat 20x/0.8 or 10x/0.45 objective (Carl Ziess AG) controlled by ZEN Black 2012 software (Carl Ziess AG); and 2) by an inverted Olympus IX83 microscope (Olympus Corporation) using an Olympus super-apochromatic 20x/0.75 objective (Olympus Corporation), captured using a Hamamatsu Flash 4.0 sCMOS camera (Hamamatsu photonics), a Spectra X(LED) light-source (Lumencore) and controlled by CellSens Dimension software (Olympus Corporation)). Post-acquisition analysis was performed using (Fiji) Image J (Schindelin et al., 2012). Briefly, nuclear segmentation was achieved using a fixed binary threshold using DAPI, the fluorescence intensity (mean grey value) of each channel was masked back to nuclei.

Flow Cytometry

Cells were dissociated using Gibco TrypLE express (Thermo Fisher Scientific) dissociation, fixed with 4% Pierce formaldehyde (w/v) methanol-free (Thermo Fisher Scientific). Cells were permeabilised in PBS with 0.5 % (v/v) Triton-X100 for 15m and blocked with PBS with 0.1 % (v/v) Triton-X100 (Sigma Aldrich), 1 % BSA fraction V (w/v) (Sigma-Aldrich, A3059). Primary incubations were completed in blocking buffer using Alexa Fluor 488 Mouse anti-SOX2 (BD Pharmingen, O30-678) and Alexa Fluor 647 Mouse anti-CDX-2 (BD Pharmingen, M39-711,). Alexa Fluor 488 Mouse IgG1 κ (MOPC-21, BD Pharmingen and Alexa Fluor 647 Mouse IgG1 κ (BD Pharmingen, MOPC-31C) were used as isotype controls. Aldehyde dehydrogenase activity was measured as per the manufacturer's guidelines using the ALDEFLUOR Kit (STEMCELL Technologies, 01700). Fluorescence was measured on a LSR II cytometer (BD Biosciences) and results were analysed using FlowJo software (FlowJo LLC).

RNA-sequencing

RNA was extracted using RNEasy mini kit (Qiagen) following the manufacturer's instructions including recommended DNase digestion step. RNA concentration was measured on a on a GloMax (Promega Corporation) and RNA integrity on TapeStation (Agilent Technologies). Libraries were prepared using KAPA mRNA (PolyA) HyperPrep Kit (Roche Holding AG, KK8581) using 500 ng RNA per sample according to manufacturer's instructions. Libraries were sequenced using a HiSeq 4000 (Illumina Biotechnology) as follows: pooled to 4 nM, 75bp single end sequencing and up to 38 million reads per sample.

RNA-seq analysis

Reads were Illumina adapter trimmed using Cutadapt v1.16 (Martin, 2011) and aligned against GRCh38 and Ensembl release 86 transcript annotations using STAR v2.5.2b (Dobin et al., 2013) via the transcript quantification software RSEM v1.3.0 (Li and Dewey, 2011). Gene-level counts were rounded to integers and subsequently used for differential expression analysis with DESeq2 (Love et al., 2014). Differential expression analysis between pairwise replicate groups was thresholded for significance based on an FDR<=0.01, a fold-change of +/- 2, and a base-mean expression of >=100. PCA analysis

was conducted on the normalised log transformed count data using the 10% most variable genes across samples. The volcano plot depicts the FDR and logFC statistics from the group DESeq2 differential expression analysis between P5 epithelial and P5 mesenchymal samples. For hierarchical clustering analysis, genes that maintained their significance and direction of change across 2 consecutive time-points were selected for visualisation in a heatmap. K-means clustering (k=10) was used to identify distinct gene clusters of related expression. Heatmaps show gene-level normalised counts, centred and scaled as z-scores. Gene ontology analysis was carried out using ToppGene Suite (ToppFun function) (Chen et al., 2009).

Comparison between data sets
Previously published Affymetrix array data were downloaded from the NCBI Gene Expression Omnibus (GEO) as GSE109267 (Frith et al., 2018). Cell files were imported into R and RMA processed using the Bioconductor package oligo with default settings. Differential expression analysis between NMP and hESC replicate groups was assessed using limma (Ritchie et al., 2015). Genes with an FDR corrected p-value <= 0.01 and fold change >= +/- 2 were called significant. NMP high genes from the Verrier et al (2018) study were provided in supplementary data and subsequently filtered using a P-value of <=0.01 (Verrier et al., 2018). The overlap between each genes list representing significantly upregulated genes at 36h was generated using BioVenn (Hulsen et al., 2008).

Quantification and statistical analysis
Statistical analyses for each experiment are described in the figure legends or in the appropriate text. In this manuscript, we define data points as independent differentiations/replicates where repeated measurements of a variable were performed on the same cell line but repeated in different experiments performed on separate occasions. Technical replicates show multiple measurements of one variable performed on the same sample within one experiment.

SUPPLEMENTAL REFERENCES

Chen, J., Bardes, E. E., Aronow, B. J. & Jegga, A. G. (2009). ToppGene Suite for gene list enrichment analysis and candidate gene prioritization. *Nucleic Acids Res.* **37,** W305-11.

Cuny, G. D., Yu, P. B., Laha, J. K., Xing, X., Liu, J.-F., Lai, C. S., Deng, D. Y., Sachidanandan, C., Bloch, K. D. & Peterson, R. T. (2008). Structure-activity relationship study of bone morphogenetic protein (BMP) signaling inhibitors. *Bioorganic & medicinal chemistry letters.* **18,** 4388-4392.

Denham, M., Hasegawa, K., Menheniott, T., Rollo, B., Zhang, D., Hough, S., Alshawaf, A., Febbraro, F., Ighaniyan, S., Leung, J., et al. (2015). Multipotent Caudal Neural Progenitors Derived from Human Pluripotent Stem Cells That Give Rise to Lineages of the Central and Peripheral Nervous System. *STEM CELLS.* **33,** 1759-1770.

Dobin, A., Davis, C. A., Schlesinger, F., Drenkow, J., Zaleski, C., Jha, S., Batut, P., Chaisson, M. & Gingeras, T. R. (2013). STAR: ultrafast universal RNA-seq aligner. *Bioinformatics.* **29,** 15-21.

Edri, S., Hayward, P., Baillie-Johnson, P., Steventon, B. J. & Martinez Arias, A. (2019). An epiblast stem cell-derived multipotent progenitor population for axial extension. *Development.* **146,** dev168187.

Frith, T. J., Granata, I., Wind, M., Stout, E., Thompson, O., Neumann, K., Stavish, D., Heath, P. R., Ortmann, D., Hackland, J. O., et al. (2018). Human axial progenitors generate trunk neural crest cells in vitro. *Elife.* **7,**

Gomez, G. A., Prasad, M. S., Wong, M., Charney, R. M., Shelar, P. B., Sandhu, N., Hackland, J. O. S., Hernandez, J. C., Leung, A. W. & Garcia-Castro, M. I. (2019). WNT/beta-catenin modulates the axial identity of embryonic stem cell-derived human neural crest. *Development.* **146,**

Gouti, M., Tsakiridis, A., Wymeersch, F. J., Huang, Y., Kleinjung, J., Wilson, V. & Briscoe, J. (2014). In vitro generation of neuromesodermal progenitors reveals distinct roles for wnt signalling in the specification of spinal cord and paraxial mesoderm identity. *PLoS Biol.* **12,** e1001937.

Hackland, J. O. S., Shelar, P. B., Sandhu, N., Prasad, M. S., Charney, R. M., Gomez, G. A., Frith, T. J. R. & Garcia-Castro, M. I. (2019). FGF Modulates the Axial Identity of Trunk hPSC-Derived Neural Crest but Not the Cranial-Trunk Decision. *Stem Cell Reports.* **12,** 920-933.

Halder, S. K., Beauchamp, R. D. & Datta, P. K. (2005). A specific inhibitor of TGF-beta receptor kinase, SB-431542, as a potent antitumor agent for human cancers. *Neoplasia (New York, N.Y.).* **7,** 509-521.

Hulsen, T., de Vlieg, J. & Alkema, W. (2008). BioVenn - a web application for the comparison and visualization of biological lists using area-proportional Venn diagrams. *BMC Genomics.* **9,** 488.

Inman, G. J., Nicolas, F. J., Callahan, J. F., Harling, J. D., Gaster, L. M., Reith, A. D., Laping, N. J. & Hill, C. S. (2002). SB-431542 is a potent and specific inhibitor of transforming growth factor-beta superfamily type I activin receptor-like kinase (ALK) receptors ALK4, ALK5, and ALK7. *Mol Pharmacol.* **62,** 65-74.

Kumamaru, H., Kadoya, K., Adler, A. F., Takashima, Y., Graham, L., Coppola, G. & Tuszynski, M. H. (2018). Generation and post-injury integration of human spinal cord neural stem cells. *Nat Methods.* **15,** 723-731.

Li, B. & Dewey, C. N. (2011). RSEM: accurate transcript quantification from RNA-Seq data with or without a reference genome. *BMC Bioinformatics.* **12,** 323.

Lippmann, E. S., Williams, C. E., Ruhl, D. A., Estevez-Silva, M. C., Chapman, E. R., Coon, J. J. & Ashton, R. S. (2015). Deterministic HOX patterning in human pluripotent stem cell-derived neuroectoderm. *Stem cell reports.* **4,** 632-644.

Martin, M. (2011). Cutadapt removes adapter sequences from high-throughput sequencing reads. *EMBnet.journal; Vol 17, No 1: Next Generation Sequencing Data AnalysisDO - 10.14806/ej.17.1.200.*

Ritchie, M. E., Phipson, B., Wu, D., Hu, Y., Law, C. W., Shi, W. & Smyth, G. K. (2015). limma powers differential expression analyses for RNA-sequencing and microarray studies. *Nucleic Acids Res.* **43,** e47.

Schindelin, J., Arganda-Carreras, I., Frise, E., Kaynig, V., Longair, M., Pietzsch, T., Preibisch, S., Rueden, C., Saalfeld, S., Schmid, B., et al. (2012). Fiji: an open-source platform for biological-image analysis. *Nature Methods.* **9,** 676-682.

Verrier, L., Davidson, L., Gierlinski, M., Dady, A. & Storey, K. G. (2018). Neural differentiation, selection and transcriptomic profiling of human neuromesodermal progenitor-like cells in vitro. *Development.* **145,**

Wang, H., Li, D., Zhai, Z., Zhang, X., Huang, W., Chen, X., Huang, L., Liu, H., Sun, J., Zou, Z., et al. (2019). Characterization and Therapeutic Application of Mesenchymal Stem Cells with Neuromesodermal Origin from Human Pluripotent Stem Cells. *Theranostics.* **9,** 1683-1697.
